# Suppressing chlorophyll degradation by silencing *OsNYC3* improves rice resistance to *Rhizoctonia solani*, the causal agent of sheath blight

**DOI:** 10.1111/pbi.13715

**Published:** 2021-10-20

**Authors:** Wenlei Cao, Huimin Zhang, Yong Zhou, Jianhua Zhao, Shuaibing Lu, Xiaoqiu Wang, Xijun Chen, Liming Yuan, Haiying Guan, Guangda Wang, Wangxin Shen, David De Vleesschauwer, Zhiqiang Li, Xiaopin Shi, Junfei Gu, Min Guo, Zhiming Feng, Zongxiang Chen, Yafang Zhang, Xuebiao Pan, Wende Liu, Guohua Liang, Changjie Yan, Keming Hu, Qiaoquan Liu, Shimin Zuo

**Affiliations:** ^1^ Key Laboratory of Plant Functional Genomics of The Ministry of Education Jiangsu Key Laboratory of Crop Genomics and Molecular Breeding Agricultural College of Yangzhou University Yangzhou China; ^2^ Jiangsu Yanjiang Institute of Agricultural Science Nantong China; ^3^ Jiangsu Key Laboratory of Crop Genetics and Physiology Jiangsu Co‐Innovation Center for Modern Production Technology of Grain Crops Yangzhou University Yangzhou China; ^4^ College of Horticulture and Plant Protection Yangzhou University Yangzhou China; ^5^ Testing Center of Yangzhou University Yangzhou China; ^6^ Maize Research Institute Shandong Academy of Agricultural Sciences National Engineering Laboratory of Wheat and Maize Key Laboratory of Biology and Genetic Improvement of Maize in Northern Yellow‐huai River Plain Ministry of Agriculture Jinan China; ^7^ Agricultural Products Group BASF Scandicci Germany; ^8^ State Key Laboratory for Biology of Plant Diseases and Insect Pests Institute of Plant Protection Chinese Academy of Agricultural Sciences Beijing China; ^9^ Joint International Research Laboratory of Agriculture and Agri‐Product Safety The Ministry of Education of China, Institutes of Agricultural Science and Technology Development Yangzhou China

**Keywords:** sheath blight, chlorophyll, resistant breeding, transcriptomics, rice (*Oryza sativa*), maize (*Zea mays*)

## Abstract

Necrotrophic fungus *Rhizoctonia solani* Kühn (*R*. *solani*) causes serious diseases in many crops worldwide, including rice and maize sheath blight (ShB). Crop resistance to the fungus is a quantitative trait and resistance mechanism remains largely unknown, severely hindering the progress on developing resistant varieties. In this study, we found that resistant variety YSBR1 has apparently stronger ability to suppress the expansion of *R*. *solani* than susceptible Lemont in both field and growth chamber conditions. Comparison of transcriptomic profiles shows that the photosynthetic system including chlorophyll biosynthesis is highly suppressed by *R*. *solani* in Lemont but weakly in YSBR1. YSBR1 shows higher chlorophyll content than that of Lemont, and inducing chlorophyll degradation by dark treatment significantly reduces its resistance. Furthermore, three rice mutants and one maize mutant that carry impaired chlorophyll biosynthesis all display enhanced susceptibility to *R*. *solani*. Overexpression of *OsNYC3*, a chlorophyll degradation gene apparently induced expression by *R*. *solani* infection, significantly enhanced ShB susceptibility in a high‐yield ShB‐susceptible variety ‘9522’. However, silencing its transcription apparently improves ShB resistance without compromising agronomic traits or yield in field tests. Interestingly, altering chlorophyll content does not affect rice resistance to blight and blast diseases, caused by biotrophic and hemi‐biotrophic pathogens, respectively. Our study reveals that chlorophyll plays an important role in ShB resistance and suppressing chlorophyll degradation induced by *R*. *solani* infection apparently improves rice ShB resistance. This discovery provides a novel target for developing resistant crop to necrotrophic fungus *R*. *solani*.

## Introduction

*Rhizoctonia solani* Kühn (*R*. *solani*) is a necrotrophic fungus that infects a very wide range of crops, including staple crops rice, wheat and maize (Molla *et al*., 2020). In rice, *R*. *solani* (*AG1 IA*) causes the sheath blight (ShB) disease, a serious worldwide rice disease posing an increasing threat due to the lack of resistant germplasms and intensive cultivation practices that favour disease development (Jia *et al*., 2009; Molla *et al*., 2020). *R*. *solani* causes lesions on both leaves and sheaths, and ultimately affects grain filling and yield in rice, resulting in yield loss up to 45% (Singh *et al*., 2019).

Comparatively, genetic improvement of rice varieties with ShB resistance is the most sustainable and effective strategy to control this disease (Helliwell and Yang, 2013). Li *et al*. (2019) recently identified *ZmFBL4* through a genome‐wide association study that regulates maize ShB resistance by strengthening lignin biosynthesis, and confirmed that this mechanism also exists in rice against *R*. *solani*. Currently, around 50 quantitative trait loci (QTLs) have been identified that confer only partial resistance to *R*. *solani* in rice (Molla *et al*., 2020). However, due to the absence of an accurate, high‐throughput phenotyping assay and ShB‐resistant germplasms, only few of these QTLs have been fine‐mapped and none are characterized so far, which has severely hindered the progress in breeding for rice ShB resistance (Jia *et al*., 2013; Singh *et al*., 2019; Zuo *et al*., 2013).

Over the past decades, studies using transgenic techniques to manipulate potential defence‐responsive genes have expanded our understanding of rice‐ *R*. *solani* interaction (Molla *et al*., 2020). Such defence‐responsive genes mainly belong to the SA or ET‐JA phytohormone signalling components, pathogenesis‐related (PR) proteins, antimicrobial peptides/compounds, secondary messengers and transcription factors (TFs). Besides these universal defence‐responsive genes in host‐pathogen interaction systems, Qiao *et al*. (2020) recently reported a rice siRNA (siR109944) and its target (*OsFBL55*) that affect rice ShB resistance via regulating auxin homeostasis. In addition, Gao *et al*. (2018) found that *R*. *solani* facilitates its nutrient acquisition by inducing expression of OsSWEET11, a sugar transporter. As expected, suppression of *OsSWEET11* enhanced rice ShB resistance and did not produce evident yield penalty (Gao *et al*., 2018). Most recently, activation of *SWEET14*‐mediated signalling by tissue‐specific expression of *DOF11* was found to increase rice ShB resistance (Kim *et al*., 2020). These results demonstrate that rice components, known as susceptible genes, are hijacked by *R*. *solani* to induce host susceptibility, and provide new insights into both rice‐ *R*. *solani* interaction mechanisms and strategies for developing ShB‐resistant varieties.

Chlorophyll, as a key compound in chloroplasts, is mostly assembled with apoproteins to form light‐harvesting complexes for light energy harvesting and conversion to chemical energy (Liu *et al*., 2004). The accumulation of adequate amounts of chlorophyll is therefore vital for plants to establish photosynthetically active chloroplasts (Jarvis and Lopez‐Juez, 2013). The chlorophyll metabolism pathways in rice have been well characterized with the isolation of chlorophyll biosynthesis (*DVR*, *YGL8*, *CAO1*, etc.) and catabolism (*NYC3*, *SGR*, etc.) genes (Peng *et al*., 2019; Yamatani *et al*., 2013; Yang *et al*., 2016). Besides its basic function in photosynthesis, some studies have demonstrated that chlorophyll also affects the defence response in plants (Kariola *et al*., 2005; Mach *et al*., 2001), because porphyrin compounds, the precursors and breakdown products of chlorophyll, are extremely phototoxic and lead to reactive oxygen species (ROS) overproduction and cell death or hypersensitive responses (Tanaka and Tanaka, 2007). Therefore, the biosynthesis and degradation of chlorophyll are highly compartmentalized and regulated. The absence of *ferredoxin‐dependent glutamate synthase1* (*Fd‐GOGAT1*), which participates in chlorophyll biosynthesis, leads to accumulation of excessive ROS levels, conferring broad‐spectrum bacterial blight resistance in rice (Chen *et al*., 2016). The chlorophyll‐deficient, *Mg‐chelatase H subunit* (*ChlH*)‐ and *phytoene desaturase* (*PDS*)‐silenced leaves accelerate hemibiotrophs *Zymoseptoria tritici*‐induced cell death in wheat (Lee *et al*., 2015). The *Cucumis sativus* mutant version of *SGR*, which is involved in chlorophyll degradation, displays a broad‐spectrum resistance against oomyceteous downy mildew, bacterial angular leaf spot and fungal anthracnose pathogens (Wang *et al*., 2018). ROS production and cell death are generally effective defence responses against infection by biotrophic pathogens, which feed on host live tissues. On the contrary, host cell death is in general beneficial to necrotrophic pathogens, which uptake nutrition from dead cells (Pitsili *et al*., 2019). Thus, the signals associated with cell death could be employed by necrotrophic pathogens to facilitate infection (Faris *et al*., 2010; Haugrud *et al*., 2019). However, available lines of evidence and our understanding are very limited.

Here, we report our new findings that chlorophyll plays an important role in regulating rice ShB resistance. We employed the resistant rice variety YSBR1 and the highly susceptible Lemont for inoculation with *R*. *solani* under natural environment conditions, and compared their transcriptomic profiles. We found that the two varieties showed a significant difference in their photosynthetic system, especially on chlorophyll metabolism in response to *R*. *solani* infection: highly suppressed in Lemont but almost no change in YSBR1. We found that YSBR1 had higher chlorophyll content than that of Lemont, and inducing chlorophyll degradation by dark treatment significantly reduces its resistance. Using rice and maize chlorophyll‐deficient mutants, we further confirmed that chlorophyll played an important role in affecting both rice and maize resistance to ShB. Subsequently, we found that *OsNYC3*, a chlorophyll degradation gene (Morita *et al*., 2009), was apparently induced expression by *R*. *solani* in a high yield ShB‐susceptible variety ‘9522’, widely planted in Jiangsu province, China. Notably, silencing *OsNYC3* significantly improves rice ShB resistance with almost no inferior effects on important agronomic traits or yield of ‘9522’ in field tests. However, the *OsNYC3* transgenic lines did not show difference on resistance to biotrophic pathogen *Xanthomonas oryzae pv. oryzae* (*Xoo*) and hemi‐biotrophic pathogen *Magnaporthe oryzae* (*M. oryzae*), which cause blight and blast diseases, respectively. This discovery provides a novel target for improving crop resistance to *R*. *solani*.

## Results

### YSBR1 shows excellent resistance to sheath blight

**Figure 1:**
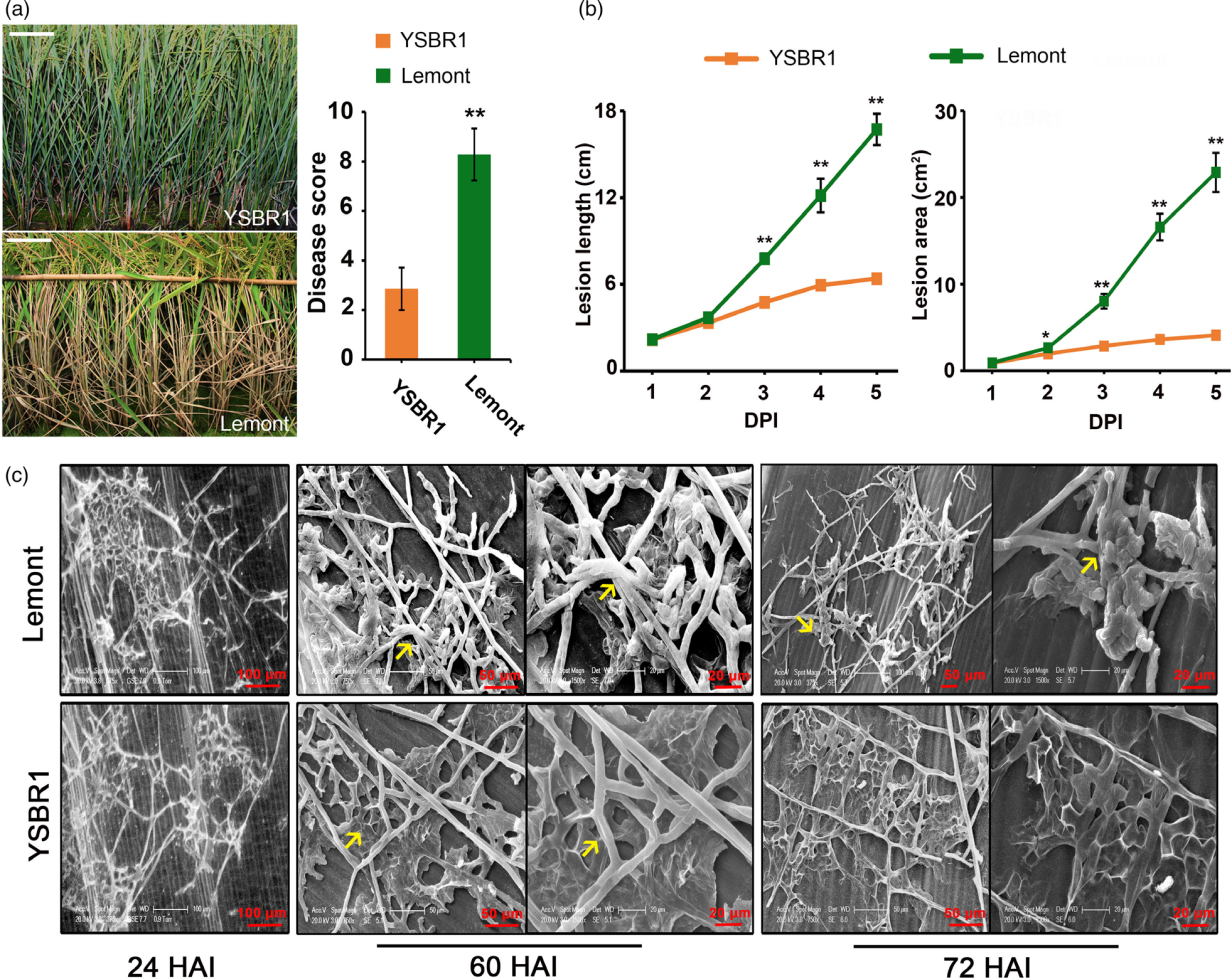
YSBR1 shows high resistance to sheath blight (ShB) with a strong ability to suppress *Rhizoctonia solani* (*R*. *solani*) infection. (a) Evaluation of YSBR1 and Lemont in resistance to ShB disease in a field. Artificial inoculation was performed at booting stage for both varieties and ShB disease scores were rated at 30 days after heading. Photographs were taken at 30 days after heading. Values are means±s.d. Scale bar, 10 cm. (b) Lesion lengths and areas of adult plants were measured at different days post inoculation (DPI) in the growth chamber. Values are means ± SD. (c) Scanning electron microscopy (SEM) of *R*. *solani* hyphal behaviour on the surface of leaf sheaths of Lemont and YSBR1 at 24, 60 and 72 h after inoculation (HAI). 8‐week‐old plants grown in natural conditions were inoculated. The inoculated sheaths were collected at the indicated time points. Infection cushions are labelled with yellow arrows. All data are presented as mean ± SD, *, *P* < 0.05, **, *P* < 0.01 using Student’s *t*‐tests.

In order to find potentially novel components that contribute to rice resistance to *R*. *solani*, we selected rice variety YSBR1, identified previously for carrying high resistance to *R*. *solani* (Zuo *et al*., 2009), and compared it to a highly susceptible variety Lemont. We first confirmed that YSBR1 is clearly more resistant to *R*. *solani* than Lemont, showing significantly lower disease scores (scores 2.86 ± 0.86 vs 8.28 ± 1.05), lesion lengths and lesion areas in both field and growth chamber tests (Figure 1a,b). We then observed *R*. *solani* infection and development on the inner sheaths, and found no clear difference on hyphae behaviour between the two varieties at 24 h after inoculation (HAI) (Figure 1c). However, at 60–72 HAI, the infection hyphae and cushions on Lemont were intact, plump and tightly tangled and closely entrenched into the inner surface of host sheaths, whereas YSBR1 contained sparse, shrivelled and chapped infection hyphae and cushions (Figure 1c). This is consistent with the data that both lesion lengths and areas showed no visible difference between the two varieties at 1 day post inoculation (DPI); while the disease lesions on YSBR1 were developed apparently slowly compared with Lemont after 2 DPI (Figure 1b). Together, these data indicate that YSBR1 is a reliable resistance variety to ShB and is able to suppress *R*. *solani* expansion after its initial infection.

### Transcriptomic analysis reveals that the known defence signals were activated in both YSBR1 and Lemont with the infection of *R*. *solani*

**Figure 2:**
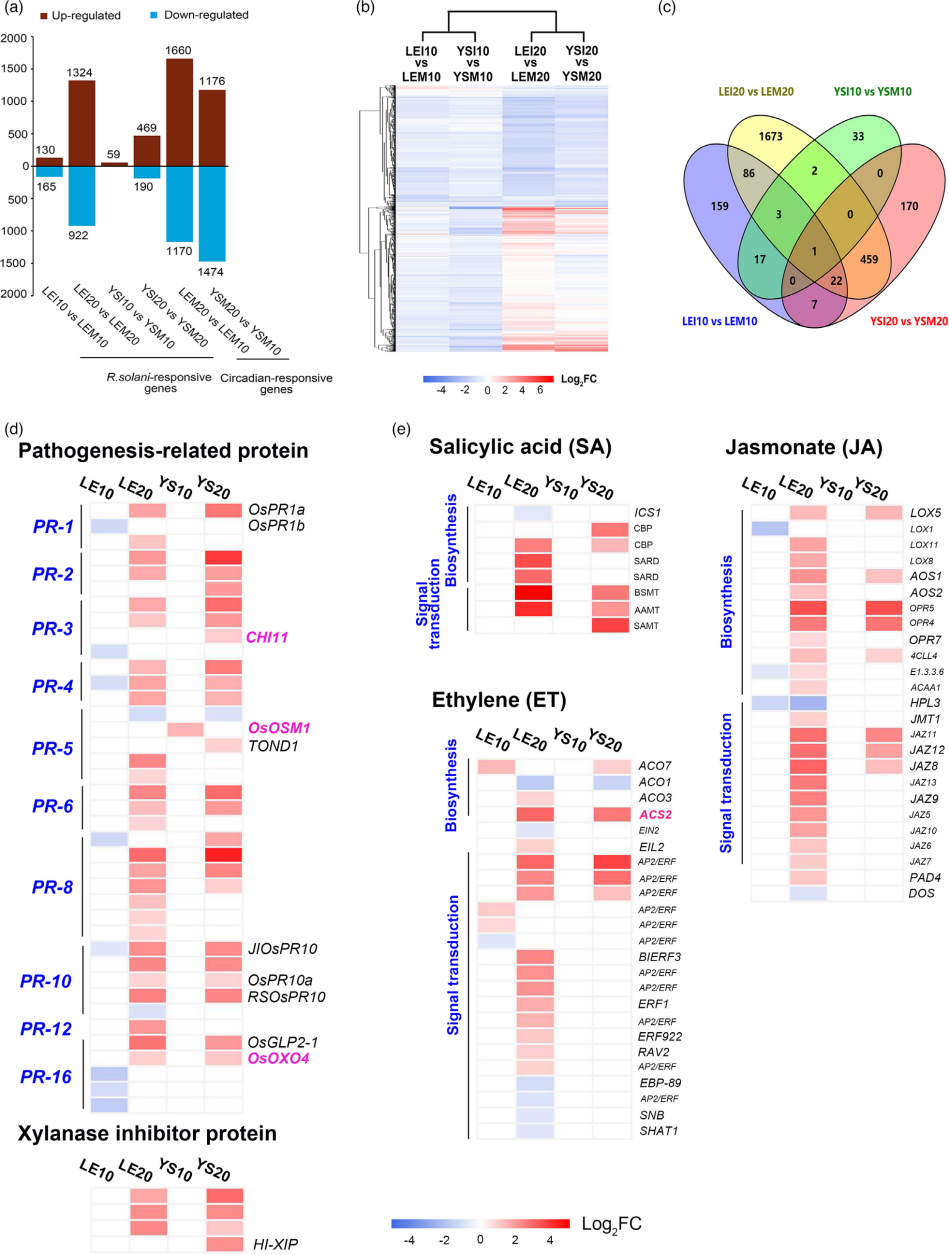
Resistant responses were activated by *Rhizoctonia solani* (*R. solani*) invasion as revealed by transcriptomics. (a) Numbers of *R. solani*‐ and circadian‐responsive genes detected in each comparison. (b) Hierarchical clustering analysis of 2632 differentially expressed genes (DEGs) in response to *R. solani*. DEGs were identified using |Log2‐Fold change (FC)| ≥0.75 and *q*‐value <0.1 as the cut‐offs. (c) Four‐way Venn diagram showing the number of common and unique *R. solani*‐responsive genes among four comparisons. (d) Comparison of YSBR1 and Lemont DEGs involved in pathogenesis‐related proteins (PRs) and xylanase inhibitor proteins (XIPs). (d) Comparison of YSBR1 and Lemont DEGs involved in pathogenesis‐related proteins (PRs) and xylanase inhibitor proteins (XIPs). (e) *R. solani* infection affects the hormone signal in both varieties. The DAVID (https://david.ncifcrf.gov/) and KEGG (https://www.kegg.jp/kegg/tool/) websites were used for gene annotation The scale bars display the Log2‐FC, shown in red when >0.75, in blue when <−0.75, and in white when −0.75 ~ 0.75.

To investigate the differential response to *R*. *solani* between YSBR1 and Lemont, we performed transcript analyses of the two varieties inoculated and un‐inoculated with *R*. *solani*. Two time‐points (10 and 20 HAI) were selected for the transcriptomic analysis using the Affymetrix Rice Genome Array. We detected over 22 000 genes expressed in leaf sheath among the 46 000 unigene models in the microarray chips. We found an excellent consistency among the three biological samples reflected by the correlation coefficient ranging from 0.9702 to 0.9954 (Table S1). Clear groupings of the 22 000 genes into rice varieties, treatments and time points support this conclusion (Figure S1a). Of these genes, we found 2632 significantly differentially expressed genes (DEGs) from the two varieties (2429 in Lemont and 714 in YSBR1) in response to *R*. *solani* infection (Figure 2a, Table S2). We randomly tested 10 DEGs using reverse transcription‐quantitative PCR (RT‐qPCR) and found a good correlation (*R* ^2^ = 0.7947) with the microarray data (Figure S1b). Noticeably, the circadian cycle caused a stronger effect on transcriptomic reprogramming than pathogen infection, because much more DEGs were found from circadian cycle (Figure 2a). Hierarchical clustering using the 2632 DEGs allowed us to conclude that the time‐point factor has the primary effect, then the variety factor on *R*. *solani* ‐induced transcriptomic reprogramming, because the 10 and 20 HAI time‐point factor separated these DEGs into two primary clusters, then the variety factor further separated the DEGs within the clusters (Figure 2b).

The two varieties shared 393 up‐regulated and 113 down‐regulated DEGs and each contained a certain number of specific DEGs (1918 in Lemont and 203 in YSBR1) (Figure 2c, Table S2). Functional category indicated that susceptible Lemont had apparently much more defence signalling related DEGs than that in YSBR1, and almost all defence signalling‐related DEGs from YSBR1 were included in Lemont DEGs with similar expression pattern (Table S3). However, the most downstream pathogenesis‐related genes (*PR*s) and xylanase inhibitor protein‐encoding genes (*XIPs*) were induced more strongly in YSBR1 than in Lemont (Figure 2d, Table S3). Some of these *PR* genes, like *OsOXO4, CHI11* and *OSM1*, have been validated as having significant effects on enhancing ShB resistance under transgenic overexpression conditions (Molla *et al*., 2020). The expression levels of *OsOXO4* significantly increased in both varieties, but *CHI11* and *OSM1* levels only increased in YSBR1 (Figure 2d). For phytohormone signals, we found most DEGs, associated with the biosynthesis and signalling of ET, JA and SA, were up‐regulated and 17 of them were detected in both Lemont and YSBR1 (Figure 2e, Table S3); in contrast, most cytokinins (CKs) and auxin‐related DEGs were down‐regulated and mainly found in Lemont (Table S3). This is consistent with previous reports showing that ET, JA and SA signals were involved in rice defence response to *R*. *solani* (Molla *et al*., 2020). For example, ET biosynthesis gene *OsACS2* significantly increased expression in both inoculated varieties (Figure 2e) and was demonstrated to enhance ShB resistance by increasing endogenous ET levels in rice (Helliwell *et al*., 2013). In addition, many DEGs encoding receptor‐like kinases or proteins (RLKs/RLPs), MAP kinases, TFs and heat shock factors/proteins (HSFs/HSPs) that are widely involved in plant defence against pathogens were activated in both varieties (Table S3). Therefore, based on these data, we conclude that the classic defence responses to *R*. *solani* are activated in both varieties although YSBR1 has much less DEGs compared with Lemont.

### Photosynthesis and antioxidant systems are clearly suppressed by *R*. *solani* in Lemont but not in YSBR1

**Figure 3:**
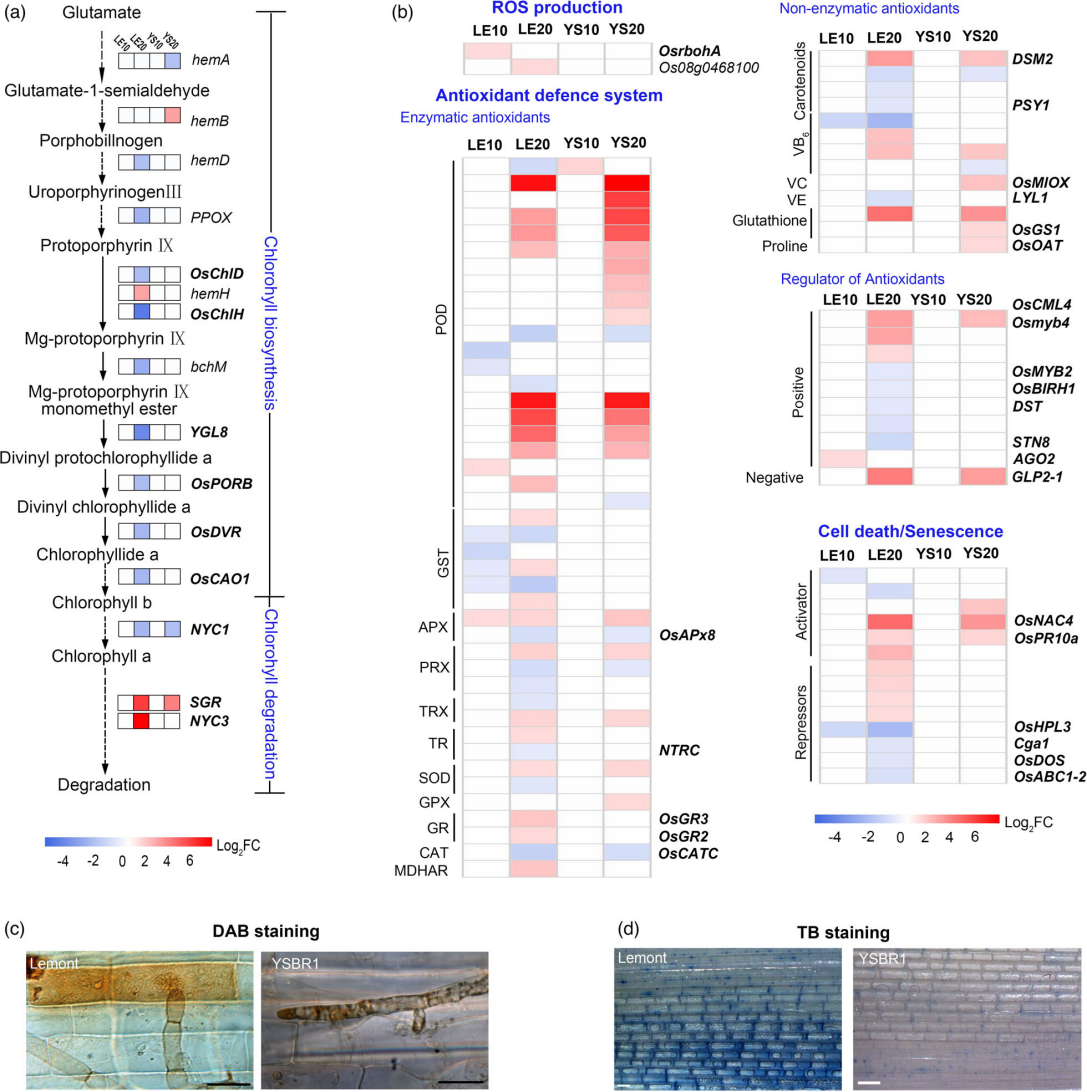
*Rhizoctonia solani* triggered *s*usceptible responses through the repression of chlorophyll and ROS metabolism. (a,b) Comparison of YSBR1 and Lemont DEGs involved in chlorophyll (a) and ROS metabolism (b). The scale bars display the Log2‐FC, shown in red when >0.75, in blue when <−0.75, and in white when −0.75 ~ 0.75. (c,d) Detection of hydrogen peroxide (H_2_O_2_) accumulation by 3,3′‐diaminobenzidine (DAB) staining (c) and cell death by trypan blue (TB) (d) in infected epidermal cells of leaf sheath at 20 HAI. Plants grown in natural conditions were inoculated at tilling stage. Scale bar, 20 μm in (c); 1 mm in (d).

Necrotrophs, such as *R*. *solani*, produce various phytotoxic metabolites or peptides to promote host cell death for their benefit (Foley *et al*., 2016). Chlorophyll loss and disintegration of photosynthetic apparatus are marker events in a cell death/senescence process (Woo *et al*., 2019). Gene ontology (GO) analysis performed for *R*. *solani*‐responsive DEGs showed that the photosynthetic system was clearly suppressed in Lemont but remained little change in YSBR1 (Figure S2). Chlorophyll metabolism was significantly disturbed in *R*. *solani*‐infected Lemont reflected by reducing the expression of chlorophyll biosynthesis genes, such as *YGL8*, *OsDVR* and *OsCAO1*, and elevating the expression of chlorophyll degradation genes, including *SGR* and *NYC3* (Figure 3a, Table S4). Moreover, we found that the down‐regulation of photosynthesis, chloroplast development and light‐responsive genes were mostly Lemont‐specific upon *R*. *solani* infection (Figure S3, Table S4). This suggests that chlorophyll is probably involved in rice response to *R*. *solani* infection.

Previous studies have demonstrated that chlorophyll reduction may accompany accumulation of ROS and reduction of host antioxidant capacity (Mur *et al*., 2010; Yang *et al*., 2016). We found that DEGs, like *RbohA* encoding respiratory burst oxidase homologs and *Os08g0468100* encoding nitrate reductase, required for ROS generation, were Lemont‐specific (Figure 3b, Table S4). The antioxidant defence system, including enzymatic and non‐enzymatic antioxidants, mainly functions to counter the accumulation of ROS for reducing its damage on cells (Pitsili *et al*., 2019). Comparatively, although the antioxidant defence system was activated by the pathogen in both varieties, it clearly showed a much stronger response in YSBR1 than in Lemont as revealed by the fold changes and numbers of POD encoding DEGs (Figure 3b, Table S4). In addition, Lemont showed evidently more down‐regulated DEGs (26 vs 55, Down‐regulated vs Total) than YSBR1 (7 vs 34) (Figure 3b and Table S4). Among them, down‐regulated genes encoding four glutathione S‐transferases (GSTs), two peroxidases and one vitamin B6 (VB6) biosynthetic enzyme involved in early response to *R*. *solani* in Lemont at 10 HAI. This implies that *R*. *solani* may effectively suppress the antioxidant defence system in Lemont but not in YSBR1, and ultimately block ROS scavenge in Lemont. Excessive ROS may lead to cell death and benefit necrotrophic infection (Pitsili *et al*., 2019). For validating the differences between the two varieties in ROS accumulation and cell death, we further conducted DAB and TB staining, and confirmed that YSBR1 had lower levels of ROS and cell death than Lemont upon *R*. *solani* infection (Figure 3b–d, Table S4).

Taken together, these data suggest that the obvious differences in chlorophyll biosynthesis/degradation and ROS production/scavenging between the two varieties are probably one of the key resistance mechanisms of YSBR1 to ShB.

### Abolishing chlorophyll biosynthesis increases rice susceptibility to *R*. *solani*

**Figure 4:**
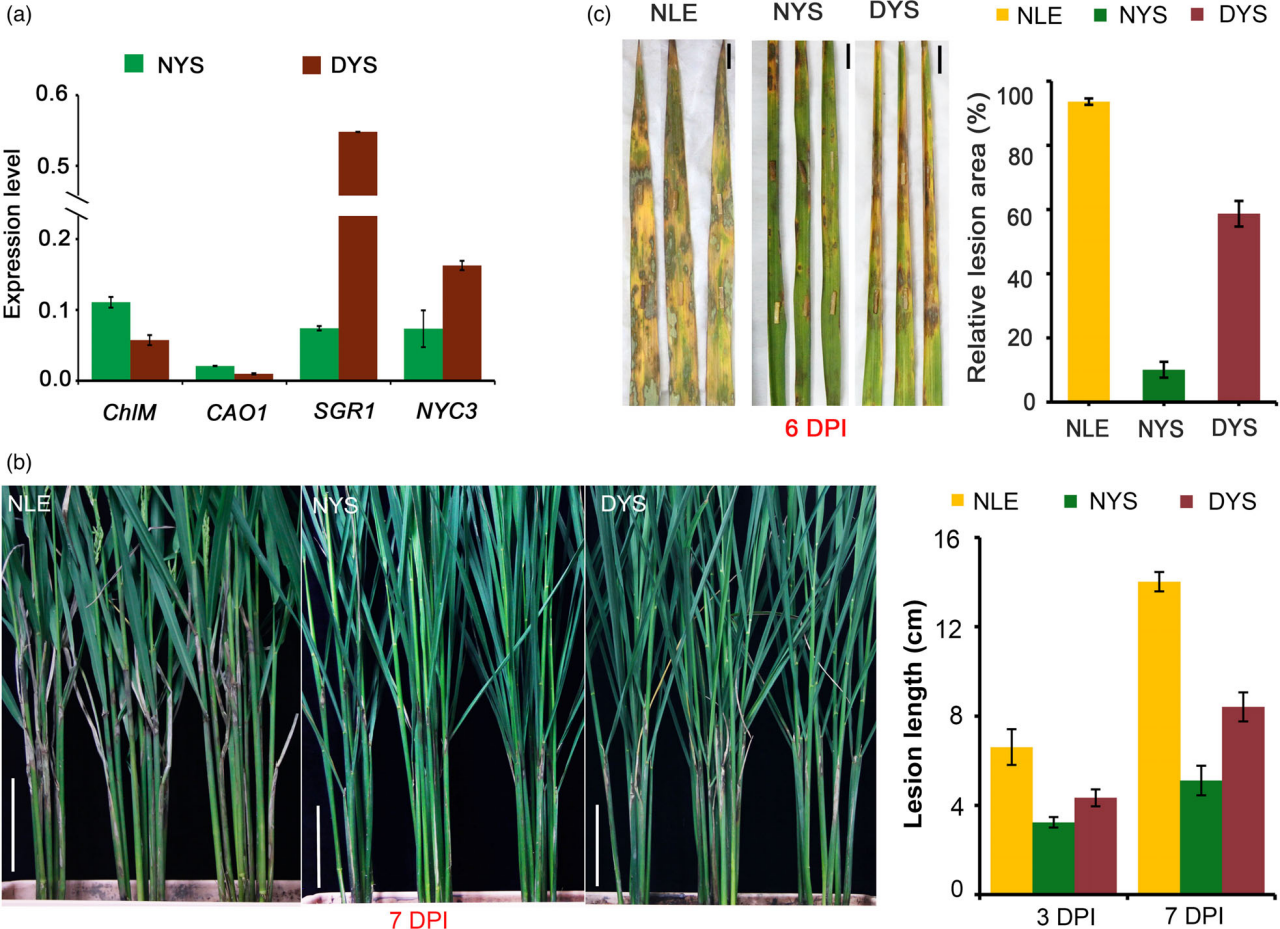
Dark treatment reduces YSBR1 resistance to *Rhizoctonia solani* (*R. solani*). (a) Changes in transcript levels of chlorophyll biosynthesis (*CHLM*, *CAO1*) and degradation (*SGR1*, *NYC3*) genes in YSBR1 untreated (NYS) and treated (DYS) with darkness. All qPCR data were normalized to *ubiquitin* (*Ubq*) and were presented as mean ± SD. The experiment was repeated three times with similar trends among each other. (b,c) The whole plants (b) and the detached leaves (c) were used for inoculation, respectively. DYS: dark‐treated YSBR1; NYS: normal YSBR1; NLE: normal Lemont. Disease symptoms were investigated at 6 DPI. Scale bar, 10 cm in (b); 1 cm in (c). Values are means ± SD.

Compared with Lemont, YSBR1 displayed more green and had significantly higher chlorophyll content in both leaf and leaf sheath at either later tillering stage or heading stage (Figure S4a). Ultrastructure observation revealed that the two varieties had no difference on chloroplast structure (Figure S4b). These implied that the high chlorophyll content of YSBR1 was not caused by the genes involved in chloroplast development but probably by the genes associated with chlorophyll metabolism. Furthermore, to empirically test the influence of chlorophyll on YSBR1 resistance to ShB, we treated YSBR1 plants with darkness to suppress chlorophyll biosynthesis and accelerate its degradation, then inoculated them with *R*. *solani*. The dark treatment significantly reduced the levels of chlorophyll biosynthesis genes (*CHLM*, *CAO1*) while up‐regulated chlorophyll degradation genes (*SGR1*, *NYC3*) (Figure 4a). We found that the dark‐treated YSBR1 plants developed lesions 1.4–1.6‐fold longer than non‐treated plants at both 3 and 7 DPI (Figure 4b). More significantly, when tested in a detached leaf assay, dark‐treated leaves developed over fivefold larger lesion areas than non‐treated leaves at 6 DPI (Figure 4c). Notably, the dark‐treated YSBR1 has reached 60%–65% of the susceptibility level of non‐treated Lemont (Figure 4b,c). These data suggest that chlorophyll degradation signals play an important role in maintaining the high ShB resistance of YSBR1.

**Figure 5:**
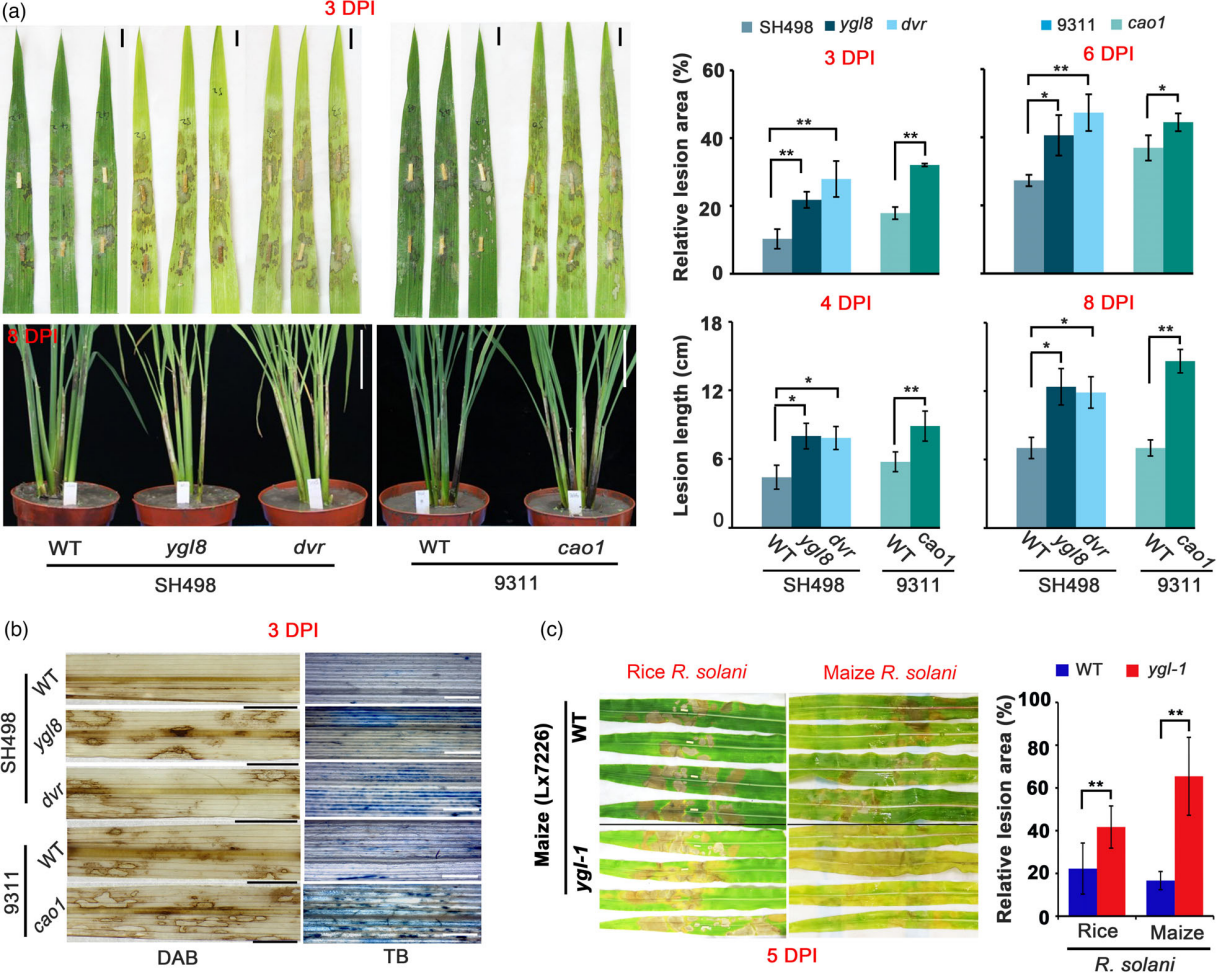
Rice and maize mutants with defects in chlorophyll biosynthesis were impaired in resistance to *Rhizoctonia solani* (*R. solani*). (a) Comparison of three rice mutants (*ygl8*, *dvr* and *cao1*) with abolished chlorophyll biosynthesis with wild type on disease symptoms post *R. solani* inoculation in detached leaf (upper) and whole plant (bottom). Pictures of inoculated leaves were taken at 3 and 6 DPI, which were used to calculate the lesion areas (upper), scale bar, 1 cm; pictures of inoculated whole plants were taken at 8 DPI, and the lesion lengths were measured at 4 and 8 DPI (bottom), scale bar, 10 cm. (b) DAB and TB staining of the three mutants and wild type at 3 DPI. Scale bar, 1 cm in left; 100 μm in right. (c) Comparison of a maize chlorophyll biosynthesis mutant *ygl‐1* with wild type on disease symptoms post *R. solani* inoculation. Scale bar, 10 cm. All data are presented as mean ± SD, *, *P* < 0.05, **, *P* < 0.01 using Student’s *t*‐tests.

To further assess the role of chlorophyll in host resistance to *R*. *solani*, we employed three chlorophyll‐biosynthesis defective rice mutants, *dvr*, *yg18* and *cao1* (Figure S5a–c). Two of the mutants carry a single‐nucleotide substitution and one has a 5‐bp deletion in the corresponding gene causing a significant reduction in chlorophyll content (Figure S5a–c). When inoculated with *R*. *solani*, we found that all 3 mutants developed lesion areas and lesion lengths significantly larger than their corresponding controls: up to twofold lesion areas at 3 DPI in detached leaf assay and around twofold lesion lengths at 8 DPI in whole plant inoculation assay (Figure 5a). Staining of infected tissues with DAB and TB showed that these mutants accumulated more ROS upon *R*. *solani* infection, resulting in greater cell death (Figure 5b). To test if maize defective in chlorophyll biosynthesis also responds similarly to *R*. *solani* infection, we inoculated a maize yellow‐green leaf mutant, *ygl‐1*, defective in chlorophyll biosynthesis (Figure S5d) (Guan *et al*., 2016), with *R*. *solani* isolated from both rice and maize. As expected, the *ygl‐1* mutant displayed lesion areas approximately twofold as large as wild type control when inoculated with the rice *R*. *solani* isolate and approximately 3.5‐fold as the control when inoculated with the maize *R*. *solani* isolate at 5 DPI (Figure 5c). These data demonstrate that chlorophyll is essential against *R*. *solani* infection in both rice and maize.

### Suppression of chlorophyll degradation by down‐regulation of *OsNYC3* gene results in enhanced resistance to *R*. *solani* with almost no detrimental effects

**Figure 6:**
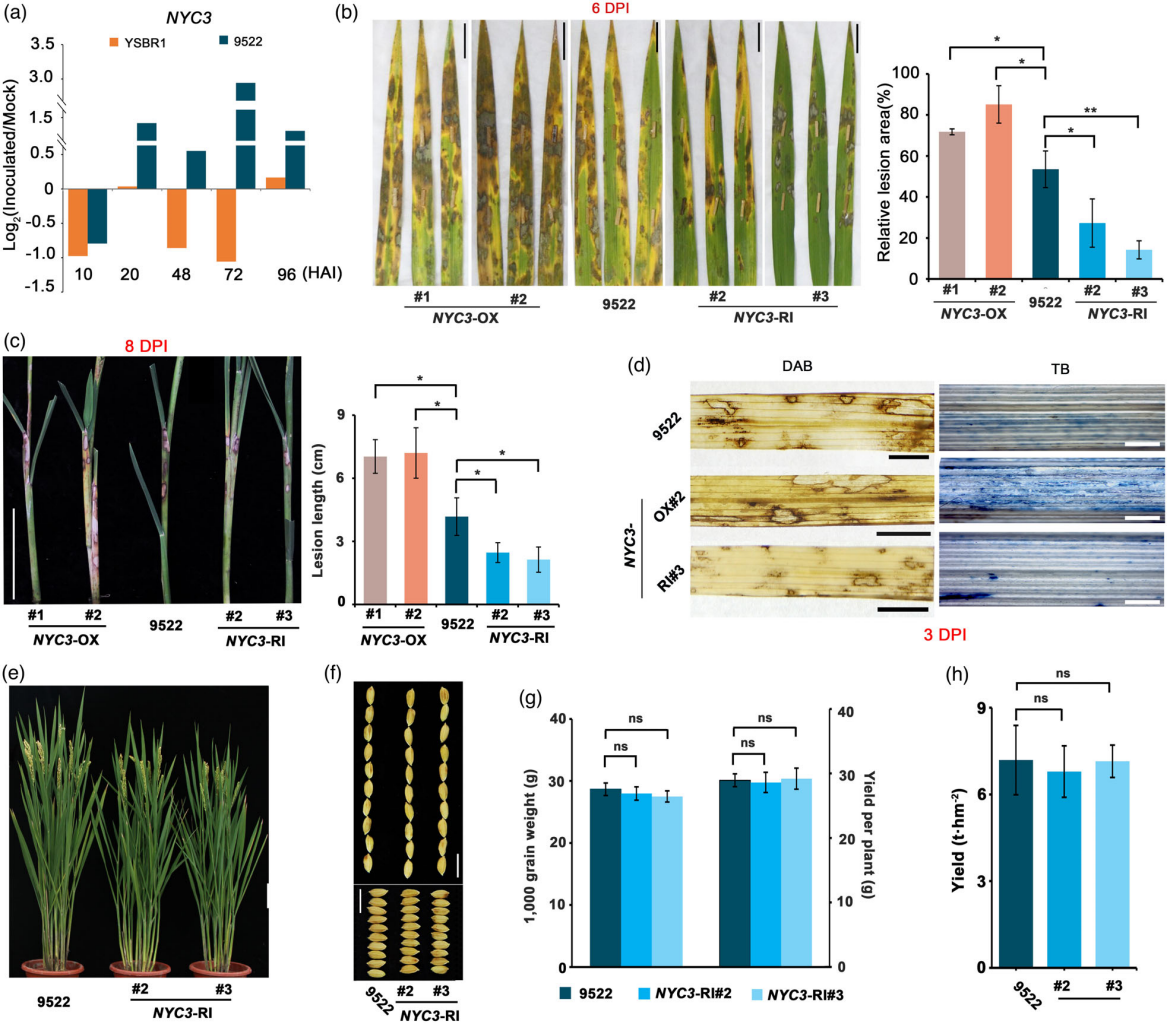
The effect of *NYC3*, a regulator of chlorophyll degradation, on rice ShB resistance and yield‐related traits. (a) *NYC3* is more significantly induced by *Rhizoctonia solani* (*R. solani*) in variety 9522 than in YSBR1. Data are shown as means ± SD. The experiment was repeated three times with similar trends among each other. (b,c) *NYC3*‐RI and *NYC3*‐OX alter rice resistance to *R. solani* in detached leaf (b) and whole plant (c) inoculations. All transgenic lines are in the 9522 variety. Images were taken and data collected at 6 and 8 DPI, respectively. Scale bar, 2 cm in (b); 5 cm in (c). (d) DAB and TB staining for H_2_O_2_ accumulation and cell death of *NYC3*‐RI, *NYC3*‐OX and wild type 9522 at 3 DPI. Scale bar, 1 cm in left, 100 μm in right. (e–h) Effects of *NYC3* silencing on the yield‐related agronomic traits. Pictures displayed the morphology of whole plant (e), grain length and width (f). (g) shown the 1000 grain weight and yield per plant, (h) shown the yield test. Scale bar, 10 cm in (e); 1 cm in (f). All data are presented as mean ± SEM; *P < 0.05; **P < 0.01; ns, not significant using Student’s *t*‐tests.

In our transcriptomic data, the *OsNYC3* (*Non‐Yellow Coloring 3*) gene, encoding a plastid‐localized α/β hydrolase‐fold family protein involved in chlorophyll degradation (Morita *et al*., 2009), was significantly induced in Lemont but not in YSBR1 (Figure 3a). We then employed qPCR to test its expression in susceptible rice variety 9522, a popular super rice (high‐yield) variety in Jiangsu, China (Figure 6a). We found that the *OsNYC3* transcription level, after an initial reduction at 10 HAI in both 9522 and YSBR1, was up‐regulated over sevenfold within 96 HAI in 9522, but was down‐regulated in most time‐points in YSBR1, indicating that *R*. *solani* may use *OsNYC3* to degrade host chlorophyll (Figure 6a). To further assess the role of *OsNYC3*, we generated two *OsNYC3* overexpression (*NYC3*‐OX) and two RNA interference (*NYC3*‐RI) transgenic lines in variety 9522. *OsNYC3* RNA levels were confirmed significantly higher in *NYC3‐OX* and lower in *NYC3‐RI* lines compared to the wild type (Figure S6a). Consistently, the *NYC3‐*OX lines displayed a distinct yellow‐green phenotype and the *NYC3‐*RI lines showed a slightly dark‐green phenotype (Figure S6b). Using detached leaf inoculation assay, we found that the *NYC3*‐OX lines displayed significantly larger (20%–40% larger) lesion areas than the wild type (WT) control whereas the *NYC3*‐RI lines displayed significantly smaller (40%–50% smaller) lesion areas (Figure 6b). Inoculation of whole plants gave similar results as detached leaf assay (Figure 6c). DAB and TB staining showed that the *NYC3‐OX* lines produced significantly more H_2_O_2_ and cell death than WT while the *NYC3‐RI* lines produced less upon *R*. *solani* infection (Figure 6d), indicating that chlorophyll probably functions to scavenge ROS, limiting cell death. Together, these data demonstrate that *R*. *solani* induces rice chlorophyll degradation to facilitate its infection, and suppression of this process improves rice ShB resistance.

Because the enhanced resistance to *R*. *solani* achieved by down‐regulation of *OsNYC3* in *NYC3*‐RI plants carries a potential value in application, we further examined these transgenic lines for their major agronomic traits in the field. We found that, except for plant height, the two *NYC3*‐RI lines shared almost the same agronomic traits, such as tiller number, seed setting rate, panicle length, grain length and width, 1000‐grain weight, with WT 9522 (Figure 6e–g, Figure S7). Most importantly, we found that these *NYC3‐*RI lines did not affect grain yield, giving similar yields as wild type 9522 in field tests (Figure 6h). For plant height, both *NYC3*‐RI lines are moderately shorter than the WT by approximately 4.0 cm (Figure S7a). Shorter rice plant height is often a preferred breeding target because it offers certain advantages, such as reduction in lodging problems. Thus, the shorter plant height trait of *NYC3*‐RI plants may be a desirable one while all other traits remain essentially unchanged. This indicates that *OsNYC3* would be a new high‐potential target for engineering ShB‐resistant varieties.

### Altering chlorophyll content not affects rice resistance to biotrophic and hemi‐biotrophic pathogens

In order to test if chlorophyll is essential for rice resistance to other pathogens, we employed *Xoo* and *M. oryzae* to inoculate two rice chlorophyll‐deficient mutants (*ygl1* and *dvr*) and *OsNYC3*‐related transgenic lines. Biotrophic pathogen *Xoo* and hemi‐biotrophic pathogen *M*. *oryzae* are the causal agent of seriously global rice diseases, blight and blast, respectively. We found that all 6 *M*. *oryzae* isolates from Jiangsu province, China, could not infect WT control SH498 and two chlorophyll‐deficient mutants in its genetic background (Figure S8a). Three of them could infect *OsNYC3* transgenic lines and its WT control 9522, but no difference on lesion size was observed among NYC3OX#1, NYC3RI#3 and the control 9522 (Figure S8a). With regard to *Xoo*, two strains were used and both could infect all plants tested, while no difference was found between all pairwise comparisons (Figure S8b). Together, these data suggest that altering chlorophyll content does not affect rice resistance to *M. oryzae* and *Xoo*.

## Discussion

### Comparing each time point with its mock control eliminates circadian genes and explains the discrepancy to previous transcriptomic data

Previously, Zhang *et al*. (2017) identified 4802 DEGs responsive to *R*. *solani* infection using Teqing and Lemont, and Yuan *et al*. (2018) reported 7624 DEGs responsive to *R*. *solani* infection. In contrast, we identified only 2632 DEGs responsive to *R*. *solani* infection, including 2429 in Lemont and 714 in YSBR1 (Table S2). Clearly the number of DEGs identified in this report is much less than previous reports. This discrepancy may be explained by our design which included more controls than previous reports as well as conducting inoculations all under natural conditions. Specifically, we included mock controls at both 10 and 20 HAI, whereas previous reports compared different *R*. *solani*‐infected time points all to time zero (Yuan *et al*., 2018; Zhang *et al*., 2017). Many of the DEGs identified in previous reports are probably not specifically induced by *R*. *solani*, but simply due to circadian rhythm. This could be validated by comparing the transcriptomic profiles of the two time points in either Lemont or YSBR1 without infection treatment. Comparing the two time points produced more than 2500 DEGs for each variety, demonstrating that circadian rhythm has a great effect on gene expression (Figure 2a). Also, our study used only leaf sheath tissues near the infected sites instead of nearly whole leaves or shoot tissues of infected plants previously reported (Karmakar *et al*., 2019; Suharti *et al*., 2016; Zhang *et al*., 2017). In addition, we grew and inoculated the plants all under natural conditions (Figure S9a), which was different from the previous studies that used a greenhouse or growth chamber (Ghosh *et al*., 2017; Karmakar *et al*., 2019; Prathi *et al*., 2018). Because of these designs, we obtained high‐quality transcriptomic data, reflected by high correlation coefficients among biological samples and RT‐qPCR validating data (Figure S1, Table S1).

Besides the above strict designs, the resistant YSBR1 used in this study should be the other reason leading to this small number of total DEGs, which produced much less DEGs than that in susceptible Lemont. At 20 HAI, most inoculation sites displayed yellowing and water‐soaked lesions in both varieties (Figure S9d), and the samples with similar diseased symptoms at this time point were collected for transcriptomic analysis. Therefore, we could exclude that much less DEGs of YSBR1 than that of Lemont at 20 HAI were caused by apparently different disease processes due to inconsistent inoculation amount or activities of inoculum. With respect to 10 HAI, however, due to no visible symptoms, we employed microscopy to observe the behaviour of infection hyphae on both varieties (Figure S9f), and which allowed us to choose the samples with similar infection behaviour of *R*. *solani* for transcriptomic analysis. Therefore, we could also exclude that the less DEGs of YSBR1 than that of Lemont at 10 HAI was caused by inconsistent inoculation amount or activities of inoculum. In addition, based on the similar growth status of six randomly selected inoculum on PDA plates (Figure S9c) and the data of cluster analysis and correlation coefficients using DEGs (Figure S1a, Table S1), we could further infer that inocula used in this study contained an almost same amount and/or activities of *R*. *solani* among each other. Together, we believe that the much less DEGs in YSBR1 than that in Lemont was due to an unknown resistant mechanism of YSBR1 to *R*. *solani*.

### Chlorophyll reduction regulated by *R*. *solani* invasion probably represents an important susceptible response of host to this necrotrophic fungus

ET, JA and SA constitute the backbone of the plant defence signalling system triggering expression of various defence genes (Pieterse *et al*., 2009), which have been widely found to regulate resistance against *R*. *solani* (Karmakar *et al*., 2017; Molla *et al*., 2020). Here, we found that all the three signalling systems were significantly activated in both YSBR1 and Lemont after inoculation, although SA signalling was generally considered a defence response against biotrophic pathogens (Figure 2e). In addition, many defence‐related DEGs encoding RLK/RLPs, calcium signalling‐related proteins, GTP‐binding proteins, MAP kinases, HSFs, HSPs, WRKY TFs and NAC TFs were detected in response to *R*. *solani* in both varieties (Table S3), which provides a valuable information for future study. PR proteins, the most downstream component of plant defence functioning to directly battle against pathogens (Helliwell and Yang, 2013). We found that, nine out of 17 groups of PR proteins displayed remarkably elevated levels in both YSBR1 and Lemont after inoculation, but the levels in YSBR1 were significantly higher than those in Lemont (Figure 2d). A similar observation was noted for xylanase inhibitor proteins (XIPs) (Figure 2d), which mainly function to inhibit pathogen xylanases for reducing their ability to degrade cell walls (Zhan *et al*., 2017). All these together allowed us to infer that YSBR1 has a slightly stronger resistance response than Lemont.

Besides the known defence‐related DEGs, in the comparison of variety‐specific DEGs, we observed a striking difference that many DEGs associated with the functions of photosystems and ROS scavenging capacity were significantly suppressed in Lemont but not or only slightly suppressed in YSBR1 (Table S4). Inhibition of chlorophyll biosynthesis and acceleration of chlorophyll degradation will reduce plant photosynthesis, promoting ROS production and cell death or senescence (Ishikawa, 2005). Several previous studies have also found that photosynthesis is apparently affected by the invasion of *R*. *solani* as well as the destruction of chloroplast (Ghosh *et al*., 2017). Considering the fact that cell death is generally required for pathogenesis of necrotrophic pathogens, we infer that suppressing these processes will favour pathogen infection through accelerating cell death, but disfavour host resistance, which could be designated as the susceptible response of the host to the pathogen. In this study, we noted that many chlorophyll biosynthetic genes were greatly suppressed and chlorophyll catabolic genes elevated in Lemont, but only slightly affected in YSBR1 (Figure 3a). Through dark treatment, we found that suppression of chlorophyll biosynthesis and activation of chlorophyll degradation could significantly reduce YSBR1 resistance (Figure 4). Subsequently, we employed three chlorophyll biosynthesis‐deficient mutants (*OsCAO1*, *OsDVR* and *OsYGL8*) and found they all displayed higher susceptibility to *R*. *solani* than the wild type (Figure 5a). We further demonstrated similar results in a maize chlorophyll biosynthesis‐deficient mutant (*ygl‐1*) (Figure 5c). Specific defects in the biosynthesis of chlorophyll have been shown to reduce the ability of ROS scavenging (Yang *et al*., 2016). We found that compared with WT after inoculation, the three chlorophyll‐deficient mutants indeed accumulated more ROS, which should be the major factor resulting in cell death (Figure 5b). In addition, the combination of omics and histochemical staining results suggest that *R*. *solani* infection clearly suppresses ROS scavenging capacity in Lemont but not in YSBR1 (Figure 3b–d). This means that YSBR1 is able to withstand ROS due to a stronger ROS‐detoxifying or scavenging capacity. Together, we hypothesize that chlorophyll is required for maintaining the high resistance of YSBR1 to ShB, which is probably associated with ROS scavenging ability.

From the side of pathogen, we could infer that *R*. *solani* manipulates chlorophyll biosynthesis and degradation, and ROS scavenging processes to induce ROS burst and cell death for its invasion. As a result, after the initial infection, we think that *R*. *solani* could not easily induce cell death through manipulating chlorophyll‐related signals in YSBR1, and which affected its acquiring nutrients from dead cells and further infection. This could account for that the sparse, shrivelled and chapped infection hyphae on YSBR1 at 60‐72 HAI but not on susceptible Lemont (Figure 1c). The lesion length and areas were very slowly developed on YSBR1 after 2 DPI, which is consistent with this abnormal hyphal morphology (Figure 1c). Besides this possibility, other factors, like the accumulation of secondary metabolites and the thickness of cell wall, may also contribute to resisting *R*. *solani* invasion, and lastly results in this kind of abnormal hyphal morphology, which is worthy of being further investigated. In addition, whether this kind of hyphae behaviour related to ShB resistance still needs further validation using many more varieties as well as the detailed observation of infection structure.

### Preventing *R*. *solani*‐induced chlorophyll reduction provides a practical tool for breeding ShB‐resistant varieties

Manipulating resistant response genes has been widely reported to improve rice ShB resistance, but few susceptible responsive genes were investigated for immunity against *R*. *solani* (Molla *et al*., 2020). Here, we have demonstrated that chlorophyll reduction is probably an important susceptible responsive of rice and maize during the infection of *R*. *solani*, and maintaining the chlorophyll content may play a universal role in protecting crops against necrotrophic pathogens like *R*. *solani*. Interestingly, we found that altering chlorophyll content did not affect rice resistance to biotrophic pathogen *Xoo* and hemi‐biotrophic pathogen *M. oryzae*, implying its specific role in crop resistance to necrotrophic pathogens. We guess this is probably due to that the immune responses in these mutants or transgenic lines are not constitutively activated, which is not like most lesion mimic mutants with constitutively activated immunity and chlorophyll‐deficient phenotype. In total, this discovery provides a novel target or strategy for developing ShB‐resistant varieties through manipulation of chlorophyll degradation‐related genes. We found that *R*. *solani* infection significantly induces expression of the *OsNYC3* gene in susceptible varieties Lemont and 9522 but not or only slightly changed in YSBR1 (Figures 3a and 6a). We then developed transgenic overexpression and RNAi lines of *OsNYC3* in the background of 9522 (Figure S6). The *OsNYC3* overexpression lines significantly enhanced ShB susceptibility and cell death after *R*. *solani* infection, while the RNAi lines evidently increased ShB resistance and reduced cell death (Figure 6b–d). More interestingly, we found that, except for a slight reduction of plant height, the *NYC3*‐RI lines are not significantly different from wild type plants on important breeding traits in field tests (Figure 6e–h, Figure S7). This means that *OsNYC3* is an excellent target for rice breeding against ShB resistance, considering that overexpression or down‐regulation of many genes generally carries detrimental effects on rice development, especially on economically important traits (Xue *et al*., 2016). Consequently, our findings provide not only a new target for developing ShB‐resistant varieties but also new insights into the interaction mechanism between crop and the necrotrophic fungus *R*. *solani*.

## Methods

### Plant materials

Rice variety YSBR1, developed by pedigree breeding from the progeny of a *japonica*/*indica* hybrid, displayed a high resistance level to rice ShB disease in both field and growth chamber (Zuo *et al*., 2009). Rice variety Lemont, a *japonica* rice cultivar from Louisiana, USA, was highly susceptible to ShB disease.

Both *ygl8* and *dvr* plants were natural mutants selected from an *indica* variety Shuihui498 (SH498). The *cao1* mutant was described by Yang *et al*. (2014). The maize mutant *ygl‐1* was described by Guan *et al*. (2016).

For the generation of the *OsNYC3*‐RI construct, the partial exon 1 and 2 of *OsNYC3* containing was amplified from *japonica* rice Nipponbare cDNA using primers RI‐F (5′‐AAAGGATCCACAATAGCAAGGCACCG‐3′) and RI‐R (5′‐AAAACTAGTA GCGAGGAGATGTAGCAG‐3′). The target fragment was then cloned into the p1022 vector and transferred into the plant binary vector p*1301UbiNOS* expressed under the control of the maize *ubiquitin* promoter. To generate the *OsNYC3*‐OX construct, the coding region of *OsNYC3* was amplified from Nipponbare cDNA using OX‐F (5′‐AAAACTAGTATGGAAGTGGTTTCTT CCAGCCACTC‐3′) and OX‐R (5′‐AAAGAGCTCTTATCTAGATATTACCCA TGTGTTGGA‐3′) inserted into the p*1301UbiNOS* vector. All the constructs were transformed into *japonica* variety 9522 via *Agrobacterium tumefaciens*‐mediated transformation.

### Culture of fungal isolate

*R*. *solani AGI‐1A* isolate RH‐9 isolated from rice (Zuo *et al*., 2013), with moderate pathogenicity, was used to inoculate rice and maize. *R*. *solani* strain YWK196 isolated from maize was used to inoculate maize (Li *et al*., 2019). The isolates were cultured on potato dextrose agar (PDA) medium at 26 °C for 3 days. Mycelial discs (*c*. 0.7 cm diameter) were transformed into potato dextrose broth (PDB) medium containing autoclaved, truncated thin toothpicks with a length of *c*. 1.0 cm at 26 °C until the mycelium twined around the toothpicks. The toothpicks with mycelia were used as the inoculum (Figure S9b). We randomly re‐cultured six inocula in fresh PDA plates and found that their growing status was very similar at 24 and 48 HAI respectively (Figure S9c), which indicates that the amount of activities of the inocula used in this study was very close.

### Sampling for microarray analysis

Rice plants were sown in pots (12 cm diameter, 20 cm high) and all grew in a pool with water under natural conditions (Figure S9a). Pesticides and fungicides were sprayed at 10 days before inoculation for ensuring plants were healthy without disease or pests. Plants at similar developmental progress, like the same tiller number and height, were selected for inoculation. A toothpick inoculum colonized with *R*. *solani* was placed at the third leaf sheath from the top, as described in detail by Zuo *et al*. (2013). Toothpicks without pathogens were used to inoculate plants as mock controls. Considering the relatively high humidity in the evening, the inoculation was conducted at 8 p.m. and kept under natural conditions.

The leaf sheath tissues at 1.0 cm away from the inoculation site with a total length of around 4.0 cm were harvested at 10 and 20 HAI for subsequent transcriptomic analysis. At 20 HAI, most inoculation sites displayed yellowing and water‐soaked lesions (Figure S9d), while no visible symptoms could be observed at 10 HAI. Therefore, to ensure the 10 HAI samples receive successful inoculation, we collected and stained its corresponding inoculation site samples by lacto‐phenol cotton blue (Figure S9e, f), and checked by microscopy for the formation of infection cushion, a specialized infection structure containing distinct hyphal aggregates (Figure S9f). Three biological replicates were conducted at 10 and 20 HAI for transcriptomic analysis, and the remaining inoculated plants were kept for confirmation of their differences in ShB resistance (Figure S9g–i).

### Transcriptome profiling and statistical analysis

Transcriptome profiling was performed according to the standard protocol of the Affymetrix Rice Genome Array (CapitalBio). Differentially expressed genes were selected using | Log_2_ Fold change | ≥0.75 and *q*‐value < 0.1 as the cut‐offs and analysed with DAVID (https://david.ncifcrf.gov), GO (http://geneontology.org) and KEGG (http://www.kegg.jp/kegg) for significant functional enrichment (Huang *et al*., 2009; Kanehisa *et al*., 2019; Mi *et al*., 2019). Heat maps were generated with R package to show gene expression levels.

### ShB fungus inoculation and disease scoring

All plants were grown in plots with sterilized soil in greenhouse till adult developmental stages (around 8 weeks), and then were transferred to growth chamber with a relative humidity of 45%–60%, a photoperiod of 13 h (30 °C) : 11 h (26 °C), light : dark for 2 days before inoculation. Five tillers in each plant were inoculated by inserting the toothpick inoculums into the third leaf sheath from the top. Lesion lengths and areas were measured with a ruler at the designed times. Disease index per variety, calculated by the average of lesion lengths or areas with five replicates per line with three plants each replicate, was used to represent the resistance level of the variety.

Field inoculation and disease scoring method used to evaluate ShB resistance at adult stage was as described by Zuo *et al*. (2013). The disease severity was rated approximately 30 days after heading. Disease index per variety, calculated by the average disease score of 30 plants from three replications, was used to represent the resistant degree.

Detached leaf inoculation method as described by Jia *et al*. (2013) was also conducted on rice and maize lines. The average of relative lesion areas of 10 leaves per line was used to represent the resistance level of the variety. The experiment was repeated three times. Disease index expressed as the relative lesion area is expressed as the ratio of Lesion area: Leaf area calculated from digital photographs using the measuring tools of Adobe Photoshop CS6 (Basu *et al*., 2016).

### Histological observation of rice in response to *R*. *solani*

Scanning electron microscope (XL‐30E SEM, Philips, Amsterdam, the Netherlands) was used for observation of the hyphal behaviour; samples were prepared as described by Sun *et al*. (2019). The lacto‐phenol cotton blue staining and preparation of inoculated sheaths at 10 HAI were performed as described by Tiwari *et al*. (2017), and light microscope (Leica DMLS, 20×‐100×) was used for observation of the hyphal development. 3,3′‐Diaminobenzidine (DAB) staining of rice mutants, transgenic lines and WT leaves was conducted using a previously described protocol (Yang *et al*., 2017). In all these experiments, the toothpick inoculum described above was used. Particularly, in DAB staining assay for YSBR1 and Lemont, we adopted mycelial suspension as the inoculum, which was obtained by resuspending the smashed mycelia with ddH_2_O. When inoculation, the mycelial suspension was injected into the leaf sheath. This kind of inoculation may lead to a much slower penetration and colonization of the tissue and more dispersedly tiny infection sites on leaf sheath compared to the toothpick inoculum covered by more intact mycelium. It allowed us to more easily compare the difference between the two varieties in response to *R*. *solani* at more early stage. After inoculation, the observation was performed as the description in De Vleesschauwer *et al*. (2006), and the images were taken with a light microscope. Cell death was visualized using trypan blue (TB) staining according to the protocol of Dietrich *et al*. (1994). At least five plants were stained at the same time for each genotype. All pictures were taken by the camera (Canon).

### RNA extraction and quantitative RT‐PCR (qRT‐PCR)

Total RNA was extracted from liquid‐nitrogen‐frozen sheath samples using the TRizol reagent (Invitrogen, Carlsbad, CA). First‐strand cDNA was synthesized from 2 μg of purified total RNA following the manufacturer's protocol (Primescript™ First‐Strand cDNA Synthesis kit; Takara, Dalian, China). RT‐qPCR was performed on CFX96^TM^ Real‐Time PCR Detection System (Bio‐Rad, Hercules, CA) using SYBR Premix ExTaq Ⅱ kit (Takara) with gene‐specific primers (Table S5). The rice *ubiquitin* (*UBQ*) transcript was used as an internal control to quantify relative transcript levels.

### Evaluation of agronomic traits in field

WT (9522) and *OsNYC3*‐RI lines were grown in fields at Yangzhou (Jiangsu) with three replicates/plots per line. Each plot occupied 30 m^2^ with 5 m in width and 6 m in length. The distances between two plants and between rows were 13.3 and 25 cm, respectively. The middle 10 plants in the central row of each plot were selected for measuring agronomic traits at the maturing stage. Various parameters of agronomic traits such as plant height, tiller number, panicle length, grain width and length, seed‐setting rate, 1000 grain weight and yield per plant. The seed‐setting rate was calculated as: (Total number of filled grains per main panicle/total number of spikelets per main panicle) × 100. Plot yields were determined when the seeds were harvested, and then normalized to yield per 10 000 m^2^ (hm^2^).

### Transmission electron microscopy

Leaves (second from the top) of 8‐week old YSBR1 and Lemont were prepared for transmission electron microscopy. The leaf segments were fixed in a 2.5% glutaraldehyde solution and then in 1% osmic acid solution. Samples were prepared as described previously (Qiu *et al*., 2018), and were observed using a JEOL JEM‐1010 electron microscope (Tokyo, Japan).

### Chlorophyll measurements

For pigment extraction, leaf tissues of 3‐week‐old *ygl8*, *dvr* and *cao1* mutants and wild‐type (SH498; 93‐11) grown in the greenhouse were homogenized and resuspended in ice‐cold 80% acetone in dim green light. Residual plant debris were removed by centrifugation. Supernatants were analysed with a visible‐light spectrophotometer and chlorophyll contents were determined using the equation of Lichtenthaler (1987). Total chlorophyll was normalized to leaf fresh weight.

### Dark treatment

YSBR1 and Lemont were grown in a growth chamber (13 h : 11 h, light : dark). Then, at the tillering stage, one‐half of the plants were treated with darkness for 3 days to suppress chlorophyll biosynthesis and induce chlorophyll degradation, and the other half of plants without darkness treatment were used as control. The experiment was repeated three times with 12 plants per replication for both dark treatment and control. For detached leaf inoculation assay, the second leaves from the top of YSBR1 and Lemont were harvested in the field. All leaves were placed on wet filter papers, and half of them were treated with darkness for 3 days. The experiment was performed three replications with 10 leaves in each replication for both dark treatment and control. After treatment, all plants or leaves were inoculated with *R*. *solani* using the method described above.

### *Magnaporthe oryzae* inoculation

*M. oryzae* isolates, JD19‐1‐1, YN670, WJA‐2, ZJB‐4, GYC‐3 and HYE‐5, were used for inoculation. All isolates were cultured on complete medium (CM) for 2 weeks in dark at 28°C, and then exposed to fluorescent lights for 1 week at room temperature for sporulation. Spore concentration was adjusted to 5 × 10^5^ spores/mL with a haemocytometer before spores were applied by punch inoculation (Park *et al*., 2012). Lesions were photographed at 7 DPI. Disease index per variety, the average lesion lengths of three replicates per line with 10 leaves per replicate, was used to represent the resistance level of the variety.

### *Xoo* inoculation

To evaluate the resistance of rice bacterial blight disease, *Xoo* strain *PX099* and *PX099a* were grown in NA media. Rice plants at the booting stage were inoculated using the leaf‐clipping method (Sun *et al*., 2004). Disease phenotype was photographed at 15 DPI. Disease index per variety, the average lesion lengths of three replicates per line with 10 leaves per replicate, was used to represent the resistance level of the variety.

## Conflicts of interest

The authors have declared no conflict of interest.

## Author contributions

S.Z. and W.C. conceived the project. W.C. analysed the data, performed most of the experiments and drafted the manuscript with other co‐authors. H.Z. performed *R*. *solani* inoculation, phenotypic scorings and histochemical staining of genetic materials; Y.Z. constructed the *NYC3*‐transgenic lines; J.Z. performed the effect of dark‐treatment on ShB resistance of YSBR1, and provided R language support; L.Y. performed the scanning electron microscopy; De Vleesschauwer performed histochemical staining. H.G generated *ygl‐1* maize mutant; X.Q and S.L. performed blast and bacterial blight inoculation; G.W., W.S., Z.L. and X.S. provided field management and phenotypic scoring; J.G conducted yield test of *NYC3*‐transgenic lines; M.G. generated *ygl8*, *dvr*, and *cao1* rice mutants; X.C., Z.F., Z.C., Y.Z., X.P., W.L., G.L. C.Y, K.H. and Q.L. supervised aspects of the project. S.Z. designed experiments, interpreted data and revised the manuscript.

## Supporting information


**Figure S1** Quality control of microarray data.
**Figure S2** Functional categories of unique genes in each variety.
**Figure S3** Photosynthesis‐related genes were responsive to *R. solani*.
**Figure S4** Difference between YSBR1 and Lemont exists in chlorophyll content, rather than chloroplast structure.
**Figure S5** Phenotypic characterization of green‐yellow leaf mutants in rice and maize.
**Figure S6** The validation of *NYC3* transgenic plants.
**Figure S7** Effects of *NYC3* silencing on other yield‐related agronomic traits.
**Figure S8** The correlation of chlorophyll with rice resistance to *Magnaporthe oryzae* (*M. oryzae*) and *Xanthomonas oryzae* pv. *oryzae* (*Xoo*).
**Figure S9** Experimental design and execution for the multi‐omics analyses.

**Table S1** The correlation coefficient between biological replicates of each detected group.
**Table S2** List of differentially expressed probes of YSBR1 and Lemont under *R. solani* infection.
**Table S3** List of differentially expressed genes (DEGs) involving with general defence response.
**Table S4** List of DEGs involving a photosynthetic and antioxidant defence system.
**Table S5** qRT‐PCR primers used in the present study.
