# Supplementary material for: Suppressing chlorophyll degradation by silencing OsNYC3 improves rice resistance to Rhizoctonia solani, the causal agent of sheath blight
Source: Plant Biotechnol J. 2021 Oct 20;20(2):335–49. doi: 10.1111/pbi.13715 (PMC8753359; doi:10.1111/pbi.13715)
Supplement: Supplementary file 1 — Figure S1 Quality control of microarray data. Figure S2 Functional categories of unique genes in each variety. Figure S3 Photosynthesis‐related genes were responsive to R. solani. Figure S4 Difference between YSBR1 and Lemont exists in chlorophyll content, rather than chloroplast structure. Figure S5 Phenotypic characterization of green‐yellow leaf mutants in rice and maize. Figure S6 The validation of NYC3 transgenic plants. Figure S7 Effects of NYC3 silencing on other yield‐related agronomic traits. Figure S8 The correlation of chlorophyll with rice resistance to Magnaporthe oryzae (M. oryzae) and Xanthomonas oryzae pv. oryzae (Xoo). Figure S9 Experimental design and execution for the multi‐omics analyses. [file PBI-20-335-s001.pptx]

## Slide 1
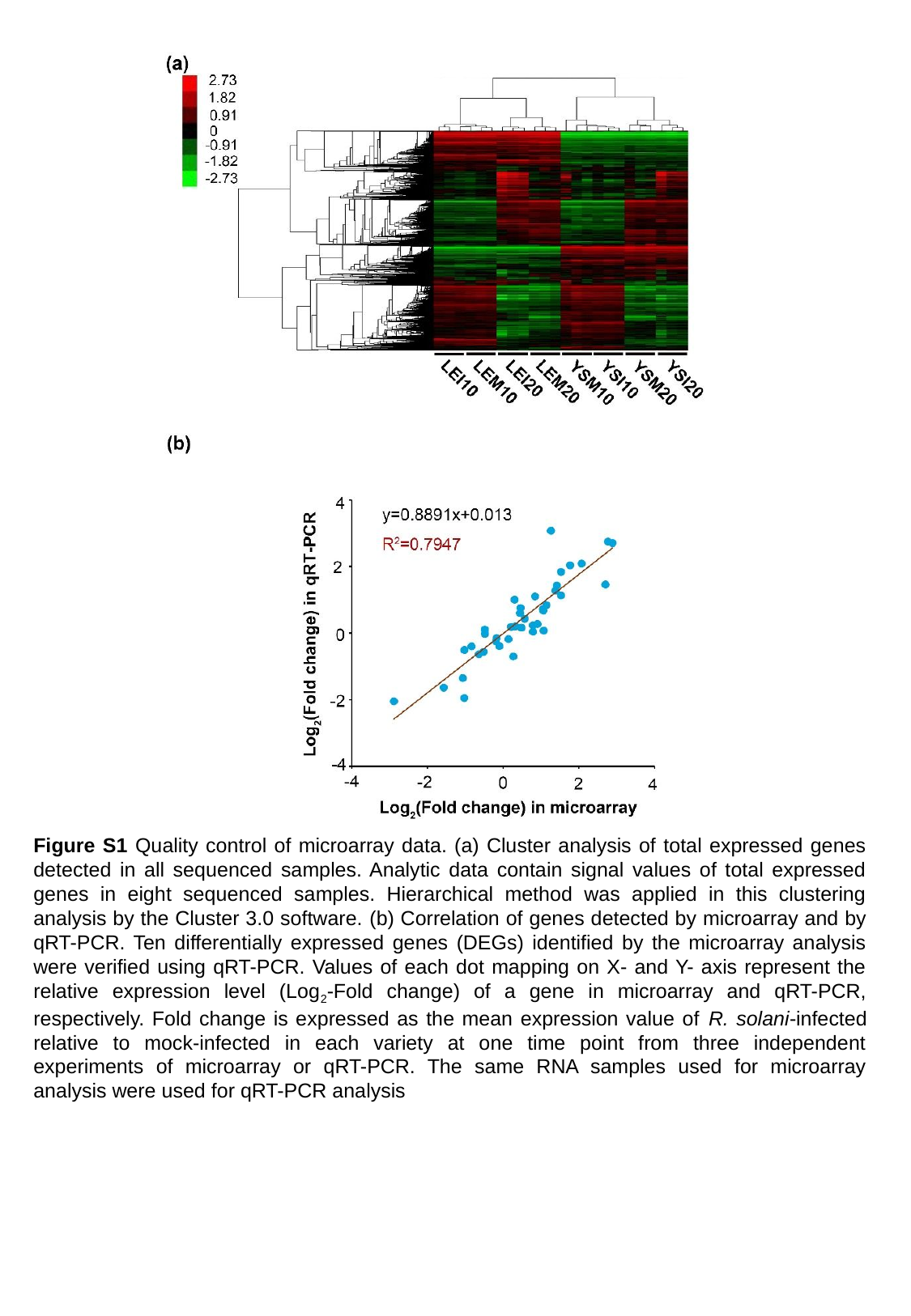

Figure S1 Quality control of microarray data. (a) Cluster analysis of total expressed genes detected in all sequenced samples. Analytic data contain signal values of total expressed genes in eight sequenced samples. Hierarchical method was applied in this clustering analysis by the Cluster 3.0 software. (b) Correlation of genes detected by microarray and by qRT-PCR. Ten differentially expressed genes (DEGs) identified by the microarray analysis were verified using qRT-PCR. Values of each dot mapping on X- and Y- axis represent the relative expression level (Log2-Fold change) of a gene in microarray and qRT-PCR, respectively. Fold change is expressed as the mean expression value of R. solani-infected relative to mock-infected in each variety at one time point from three independent experiments of microarray or qRT-PCR. The same RNA samples used for microarray analysis were used for qRT-PCR analysis

## Slide 2
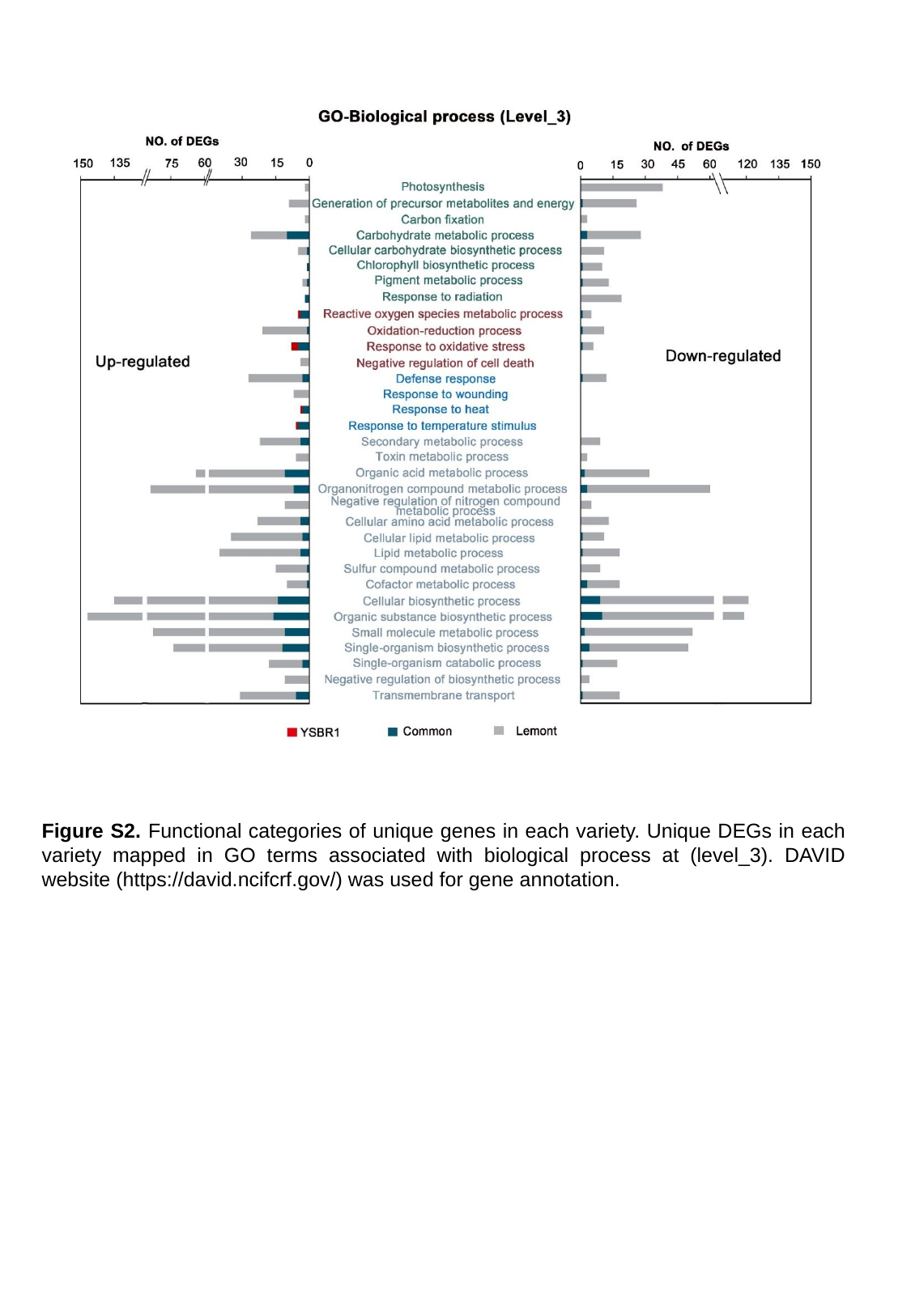

Figure S2. Functional categories of unique genes in each variety. Unique DEGs in each variety mapped in GO terms associated with biological process at (level_3). DAVID website (https://david.ncifcrf.gov/) was used for gene annotation.

## Slide 3
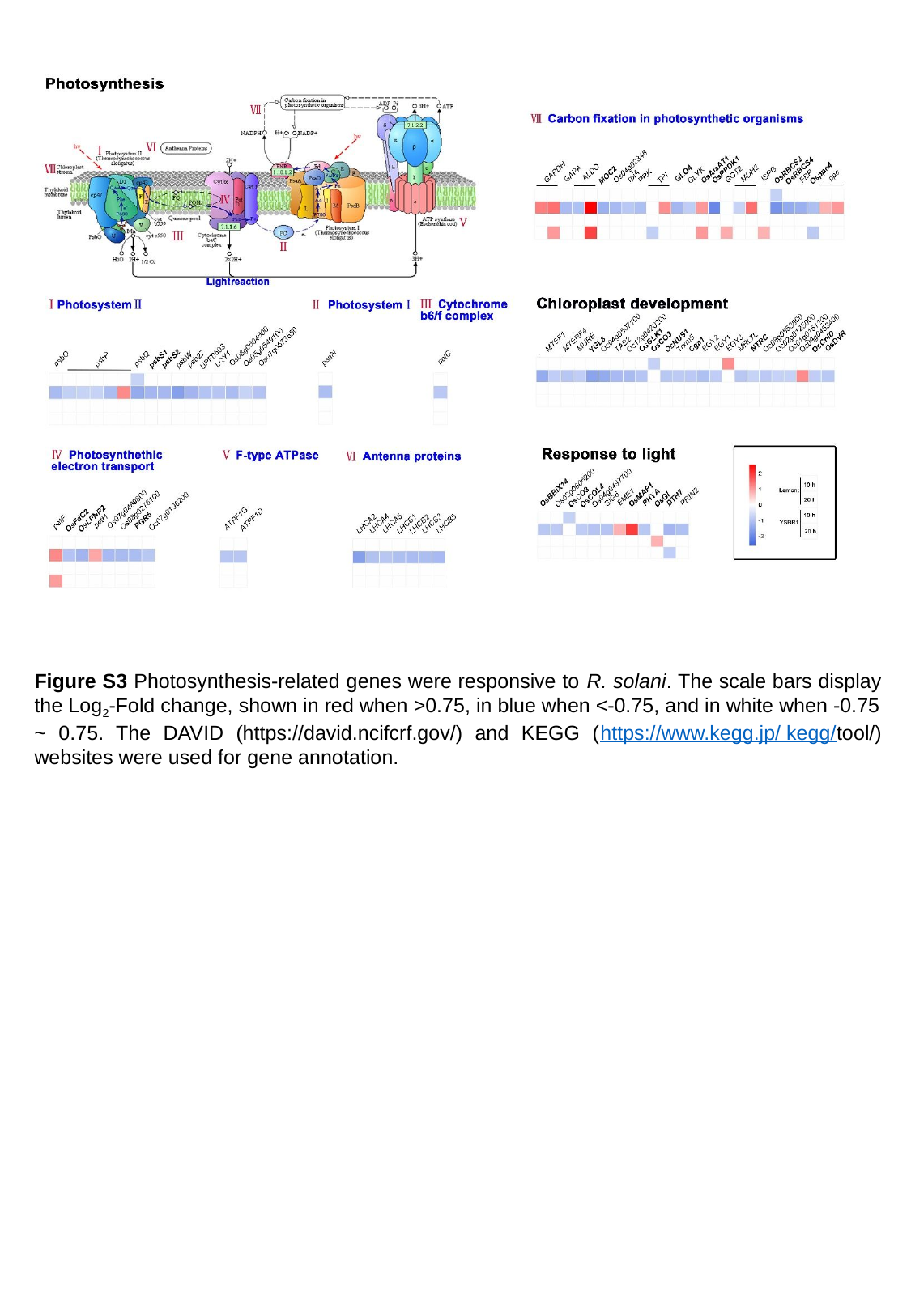

Figure S3 Photosynthesis-related genes were responsive to R. solani. The scale bars display the Log2-Fold change, shown in red when >0.75, in blue when <-0.75, and in white when -0.75 ~ 0.75. The DAVID (https://david.ncifcrf.gov/) and KEGG (https://www.kegg.jp/ kegg/tool/) websites were used for gene annotation.

## Slide 4
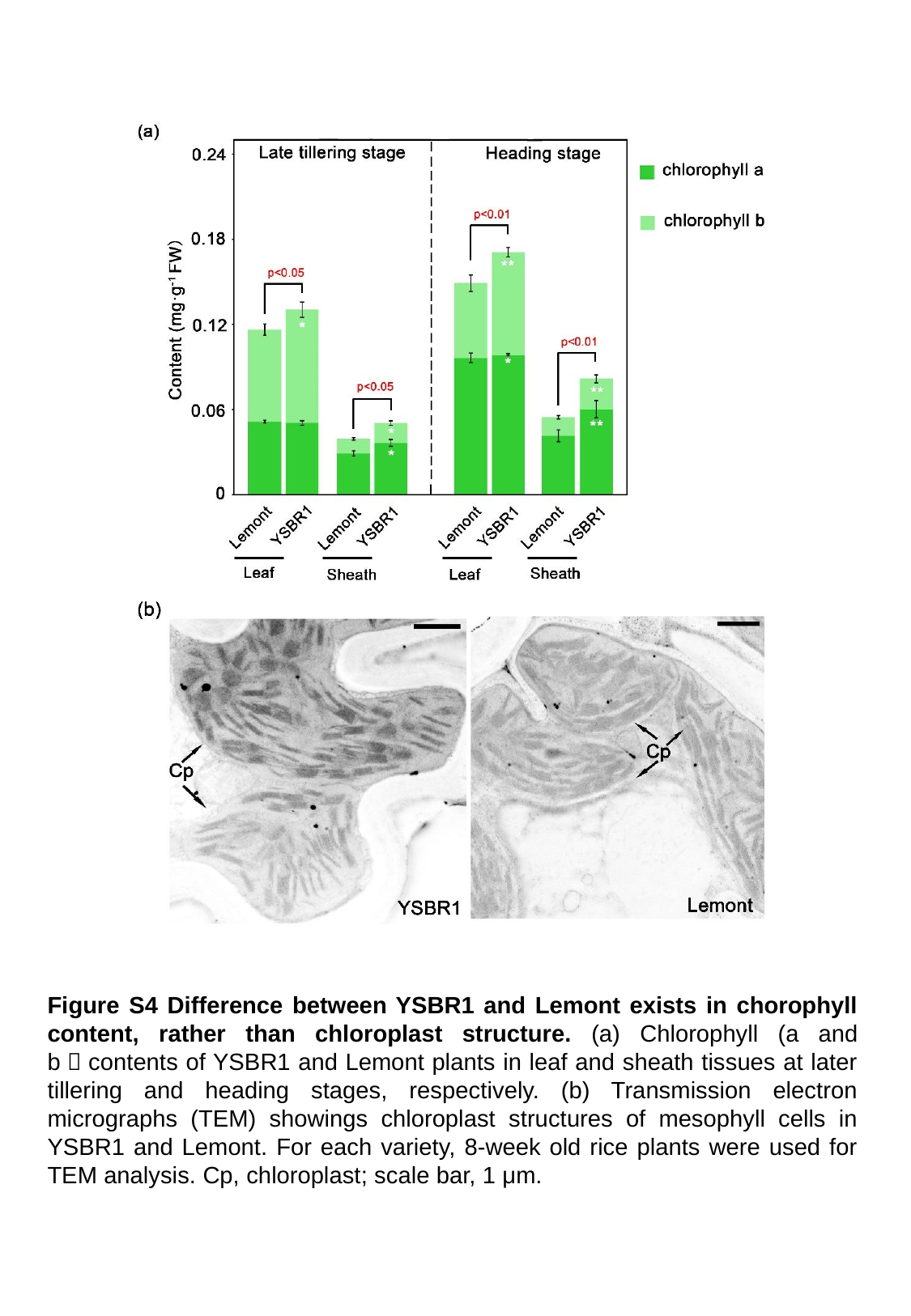

Figure S4 Difference between YSBR1 and Lemont exists in chorophyll content, rather than chloroplast structure. (a) Chlorophyll (a and b）contents of YSBR1 and Lemont plants in leaf and sheath tissues at later tillering and heading stages, respectively. (b) Transmission electron micrographs (TEM) showings chloroplast structures of mesophyll cells in YSBR1 and Lemont. For each variety, 8-week old rice plants were used for TEM analysis. Cp, chloroplast; scale bar, 1 μm.

## Slide 5
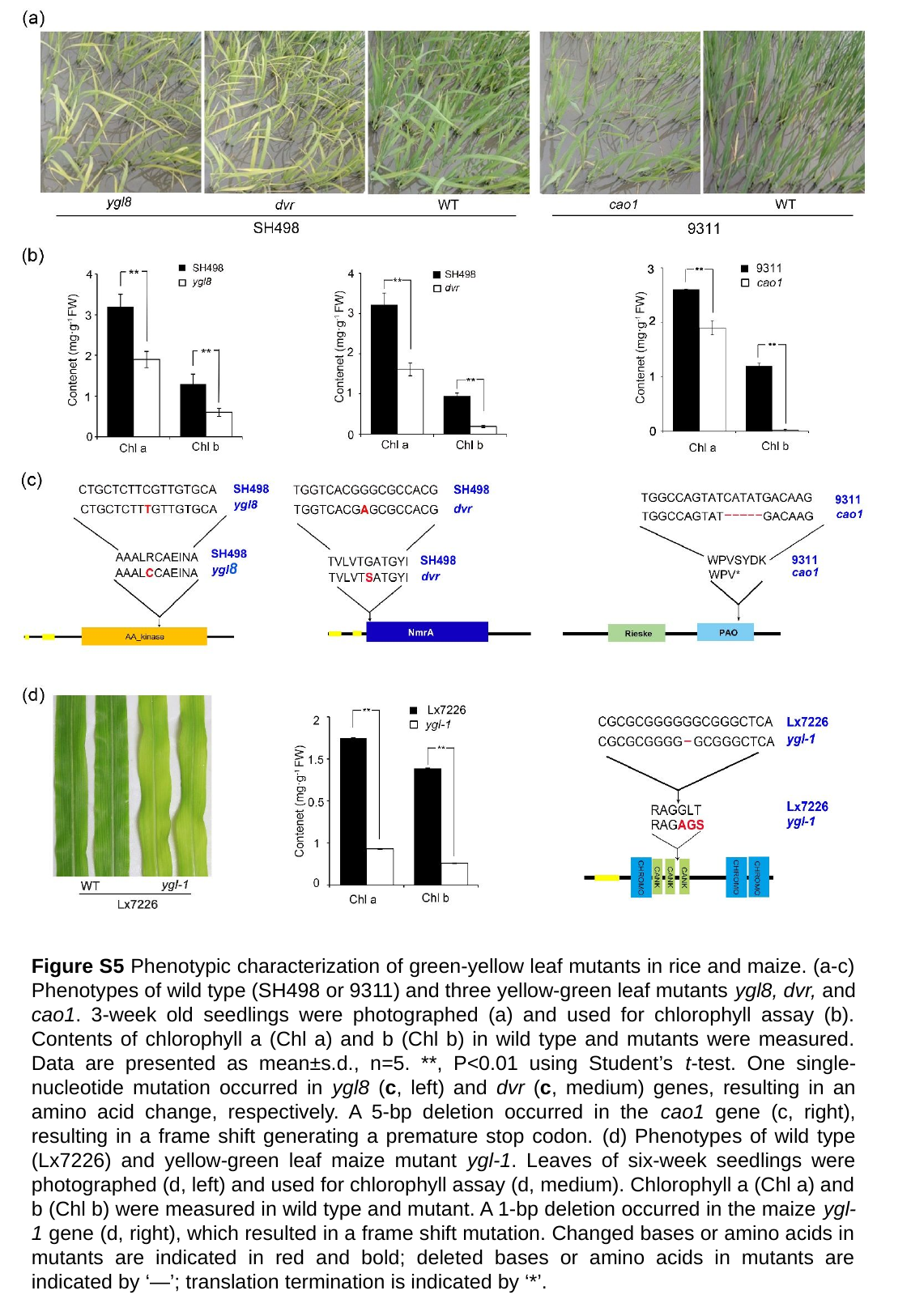

Figure S5 Phenotypic characterization of green-yellow leaf mutants in rice and maize. (a-c) Phenotypes of wild type (SH498 or 9311) and three yellow-green leaf mutants ygl8, dvr, and cao1. 3-week old seedlings were photographed (a) and used for chlorophyll assay (b). Contents of chlorophyll a (Chl a) and b (Chl b) in wild type and mutants were measured. Data are presented as mean±s.d., n=5. **, P<0.01 using Student’s t-test. One single-nucleotide mutation occurred in ygl8 (c, left) and dvr (c, medium) genes, resulting in an amino acid change, respectively. A 5-bp deletion occurred in the cao1 gene (c, right), resulting in a frame shift generating a premature stop codon. (d) Phenotypes of wild type (Lx7226) and yellow-green leaf maize mutant ygl-1. Leaves of six-week seedlings were photographed (d, left) and used for chlorophyll assay (d, medium). Chlorophyll a (Chl a) and b (Chl b) were measured in wild type and mutant. A 1-bp deletion occurred in the maize ygl-1 gene (d, right), which resulted in a frame shift mutation. Changed bases or amino acids in mutants are indicated in red and bold; deleted bases or amino acids in mutants are indicated by ‘—’; translation termination is indicated by ‘*’.

## Slide 6
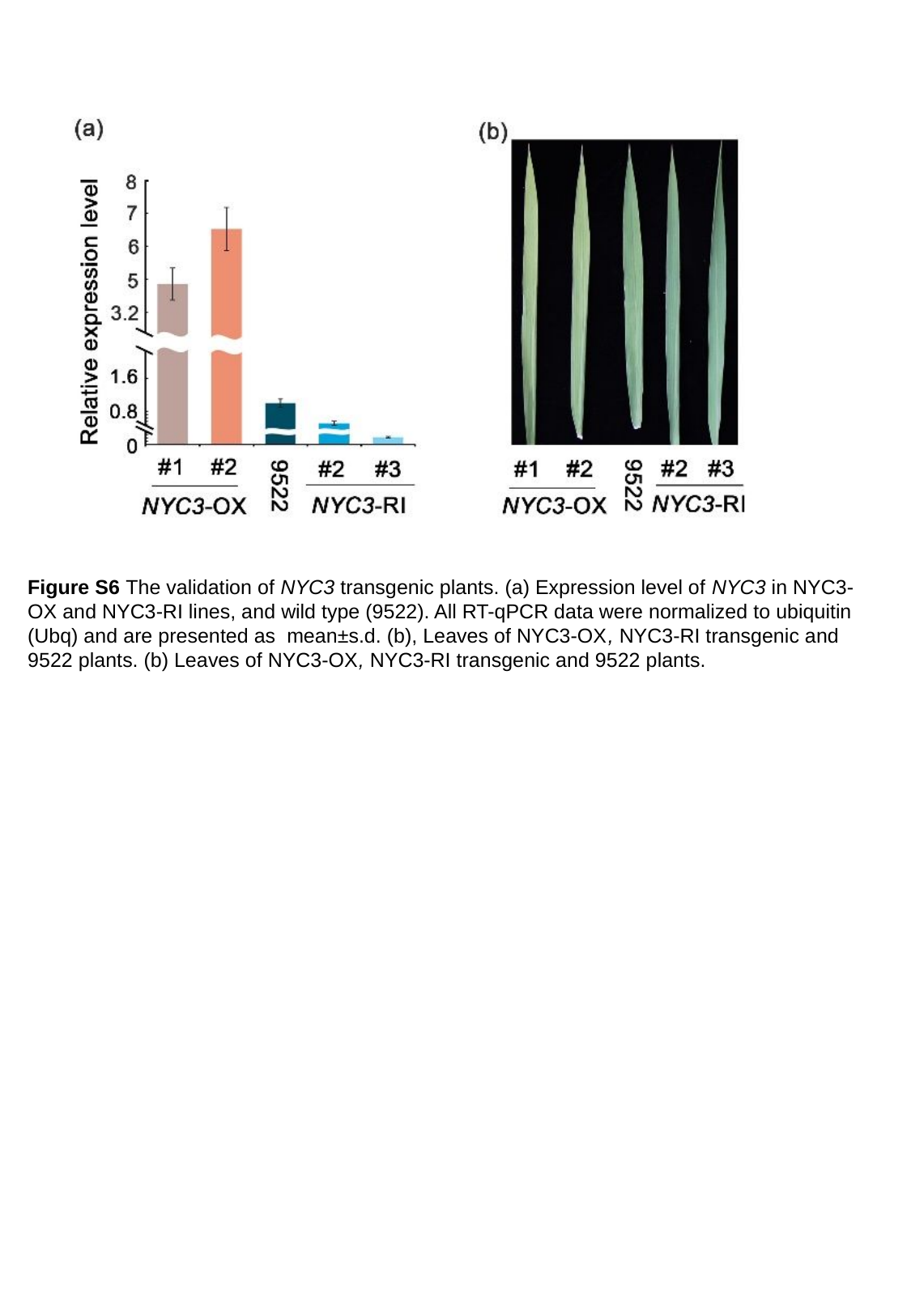

Figure S6 The validation of NYC3 transgenic plants. (a) Expression level of NYC3 in NYC3-OX and NYC3-RI lines, and wild type (9522). All RT-qPCR data were normalized to ubiquitin (Ubq) and are presented as mean±s.d. (b), Leaves of NYC3-OX, NYC3-RI transgenic and 9522 plants. (b) Leaves of NYC3-OX, NYC3-RI transgenic and 9522 plants.

## Slide 7
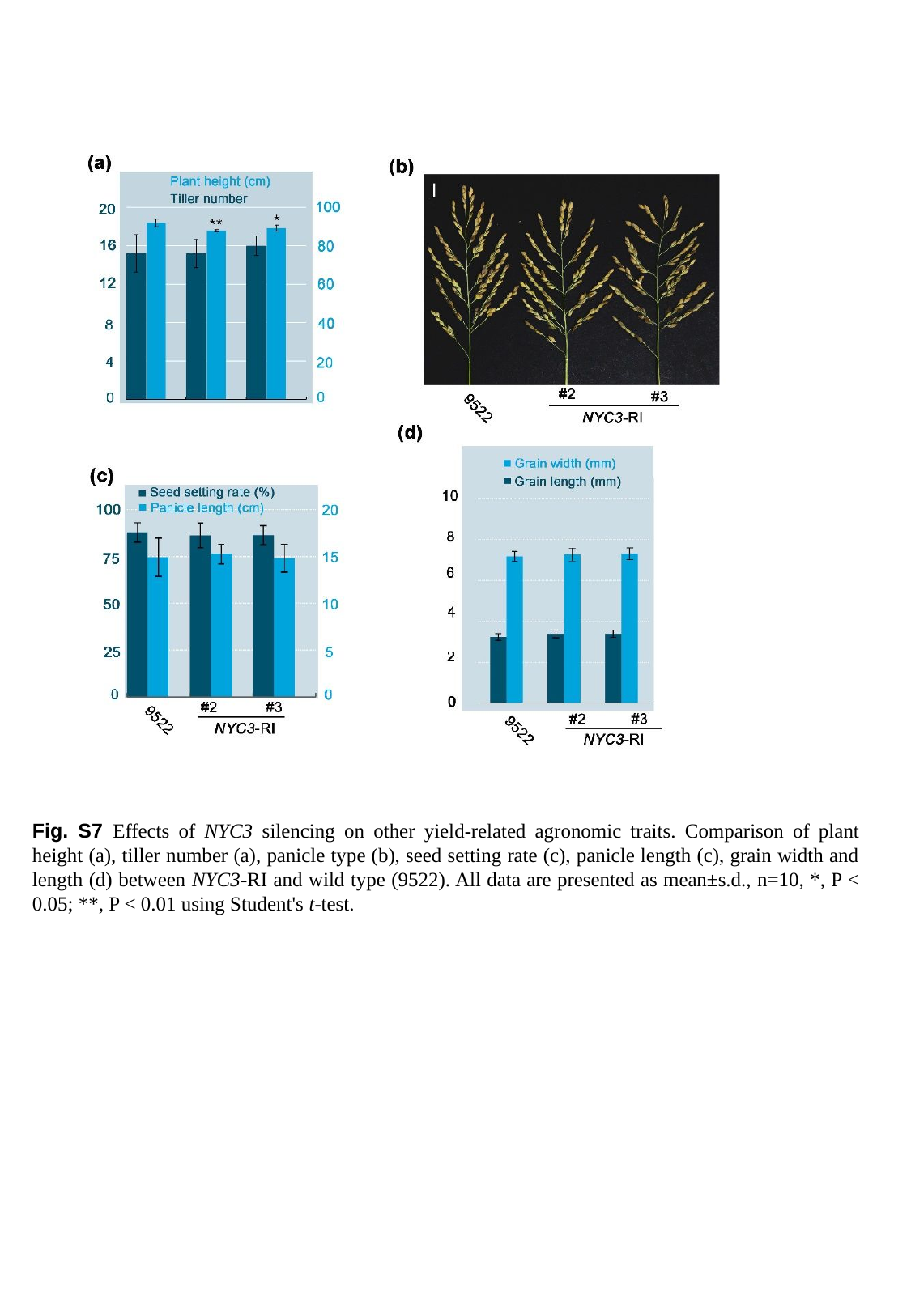

Fig. S7 Effects of NYC3 silencing on other yield-related agronomic traits. Comparison of plant height (a), tiller number (a), panicle type (b), seed setting rate (c), panicle length (c), grain width and length (d) between NYC3-RI and wild type (9522). All data are presented as mean±s.d., n=10, *, P < 0.05; **, P < 0.01 using Student's t‐test.

## Slide 8
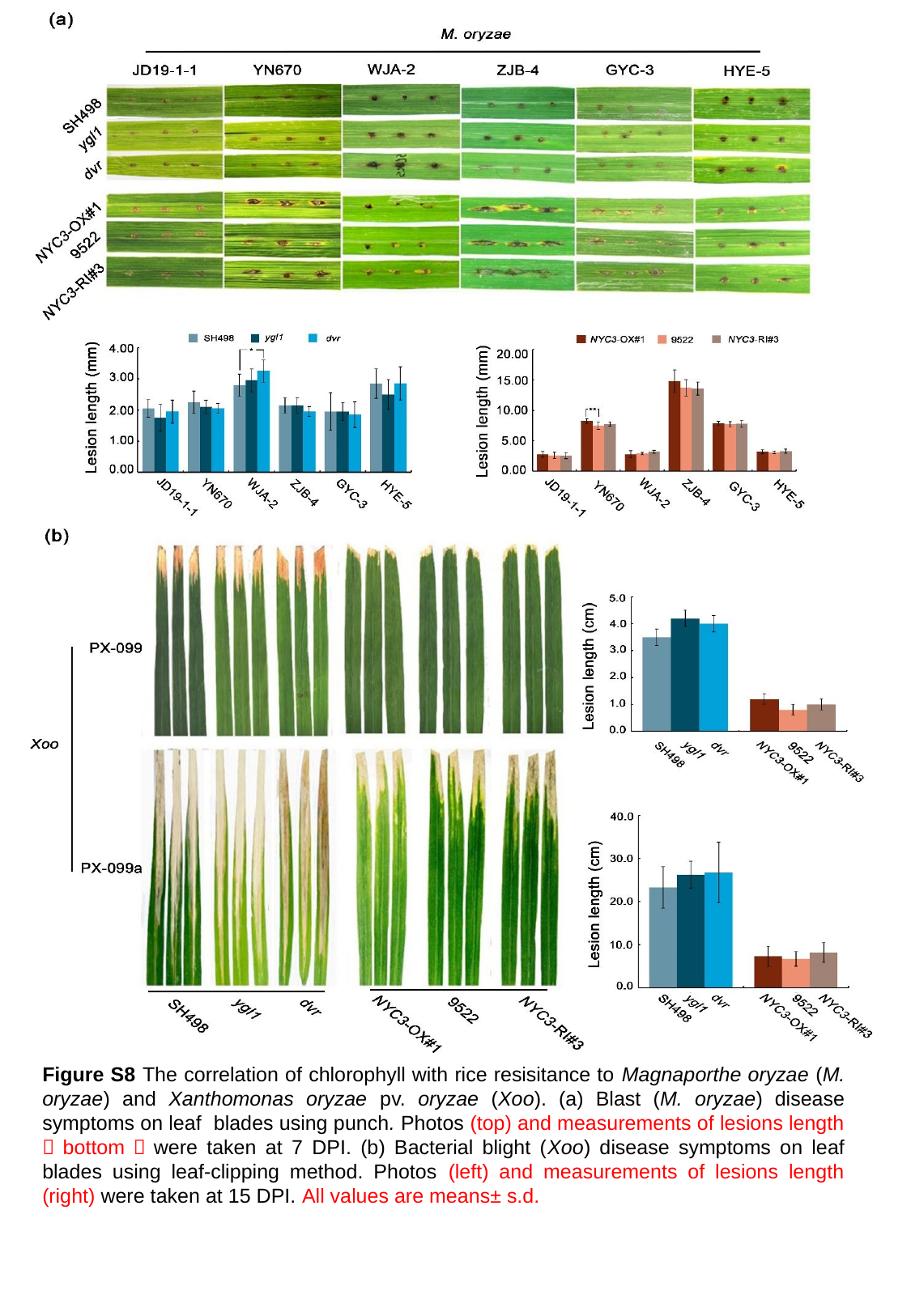

Figure S8 The correlation of chlorophyll with rice resisitance to Magnaporthe oryzae (M. oryzae) and Xanthomonas oryzae pv. oryzae (Xoo). (a) Blast (M. oryzae) disease symptoms on leaf blades using punch. Photos (top) and measurements of lesions length （bottom）were taken at 7 DPI. (b) Bacterial blight (Xoo) disease symptoms on leaf blades using leaf-clipping method. Photos (left) and measurements of lesions length (right) were taken at 15 DPI. All values are means± s.d.

## Slide 9
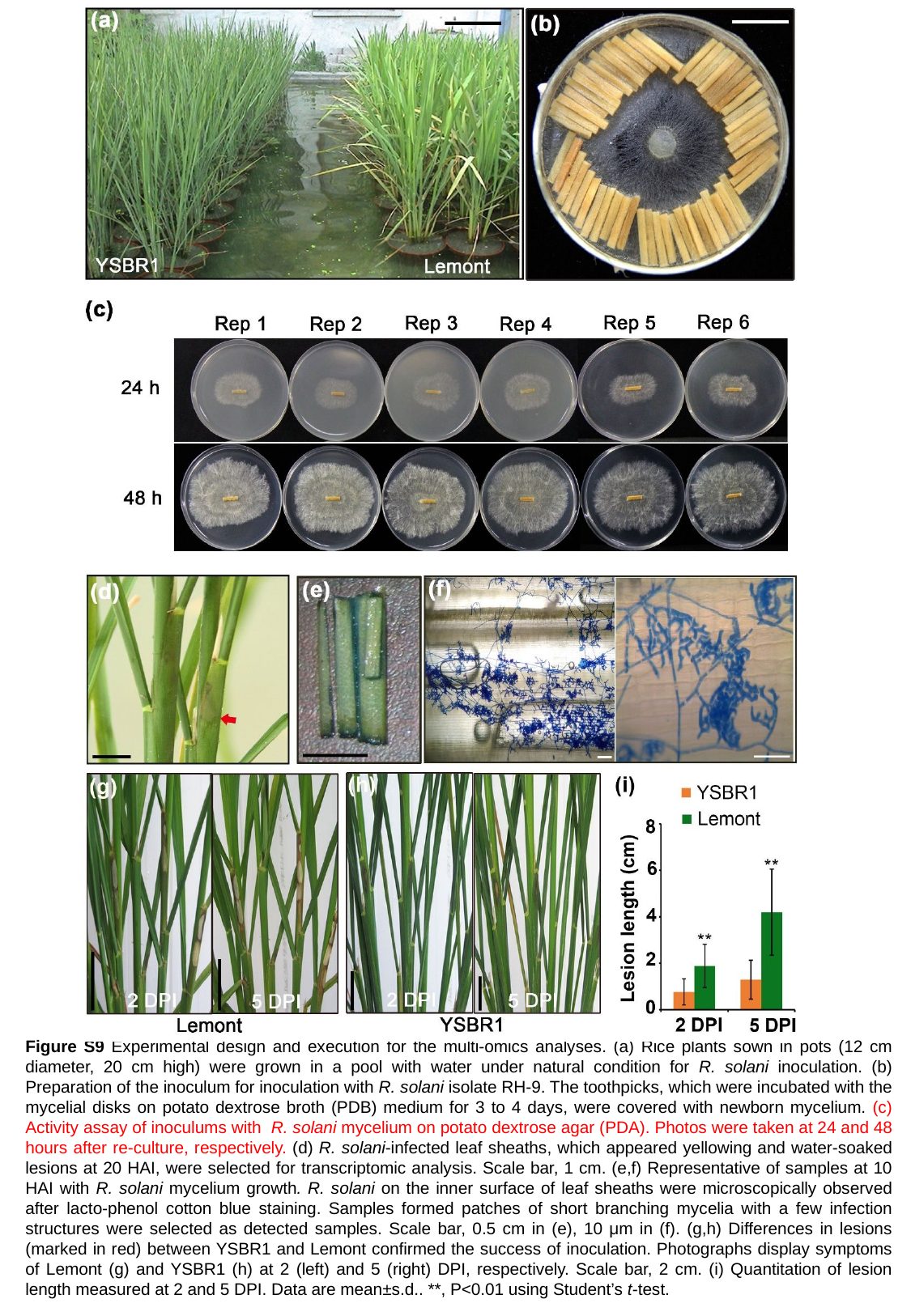

Figure S9 Experimental design and execution for the multi-omics analyses. (a) Rice plants sown in pots (12 cm diameter, 20 cm high) were grown in a pool with water under natural condition for R. solani inoculation. (b) Preparation of the inoculum for inoculation with R. solani isolate RH-9. The toothpicks, which were incubated with the mycelial disks on potato dextrose broth (PDB) medium for 3 to 4 days, were covered with newborn mycelium. (c) Activity assay of inoculums with R. solani mycelium on potato dextrose agar (PDA). Photos were taken at 24 and 48 hours after re-culture, respectively. (d) R. solani-infected leaf sheaths, which appeared yellowing and water-soaked lesions at 20 HAI, were selected for transcriptomic analysis. Scale bar, 1 cm. (e,f) Representative of samples at 10 HAI with R. solani mycelium growth. R. solani on the inner surface of leaf sheaths were microscopically observed after lacto-phenol cotton blue staining. Samples formed patches of short branching mycelia with a few infection structures were selected as detected samples. Scale bar, 0.5 cm in (e), 10 μm in (f). (g,h) Differences in lesions (marked in red) between YSBR1 and Lemont confirmed the success of inoculation. Photographs display symptoms of Lemont (g) and YSBR1 (h) at 2 (left) and 5 (right) DPI, respectively. Scale bar, 2 cm. (i) Quantitation of lesion length measured at 2 and 5 DPI. Data are mean±s.d.. **, P<0.01 using Student’s t-test.
